# Adaptive phenotypic plasticity in a clonal invader

**DOI:** 10.1002/ece3.4009

**Published:** 2018-04-02

**Authors:** Gerlien Verhaegen, Kyle E. McElroy, Laura Bankers, Maurine Neiman, Martin Haase

**Affiliations:** ^1^ AG Vogelwarte Zoological Institute and Museum Ernst‐Moritz‐Arndt University of Greifswald Greifswald Germany; ^2^ Department of Biology The University of Iowa Iowa City IA USA

**Keywords:** genetic adaptation, geometric morphometrics, invasion, phenotypic plasticity, *Potamopyrgus antipodarum*, shape, size

## Abstract

Organisms featuring wide trait variability and occurring in a wide range of habitats, such as the ovoviviparous New Zealand freshwater snail *Potamopyrgus antipodarum*, are ideal models to study adaptation. Since the mid‐19th century, *P. antipodarum*, characterized by extremely variable shell morphology, has successfully invaded aquatic areas on four continents. Because these obligately and wholly asexual invasive populations harbor low genetic diversity compared to mixed sexual/asexual populations in the native range, we hypothesized that (1) this phenotypic variation in the invasive range might be adaptive with respect to colonization of novel habitats, and (2) that at least some of the variation might be caused by phenotypic plasticity. We surveyed 425 snails from 21 localities across northwest Europe to attempt to disentangle genetic and environmental effects on shell morphology. We analyzed brood size as proxy for fitness and shell geometric morphometrics, while controlling for genetic background. Our survey revealed 10 SNP genotypes nested into two mtDNA haplotypes and indicated that mainly lineage drove variation in shell shape but not size. Physicochemical parameters affected both shell shape and size and the interaction of these traits with brood size. In particular, stronger stream flow rates were associated with larger shells. Our measurements of brood size suggested that relatively larger slender snails with relatively large apertures were better adapted to strong flow than counterparts with broader shells and relatively small apertures. In conclusion, the apparent potential to modify shell morphology plays likely a key role in the invasive success of *P. antipodarum*; the two main components of shell morphology, namely shape and size, being differentially controlled, the former mainly genetically and the latter predominantly by phenotypic plasticity.

## 1 INTRODUCTION

There is an increasingly urgent need to understand and predict how organisms will cope with the environmental consequences of global climate change (e.g., Chevin, Lande, & Mace, 2010; Dawson, Jackson, House, Prentice, & Mace, 2011; Salamin, Wüest, Lavergne, Thuiller, & Pearman, 2010). Organisms can deal with changing conditions in two ways: stay and adapt or move to a more suitable habitat (Gienapp, Teplitsky, Alho, Mills, & Merilä, 2008; Salamin et al., 2010). The latter is not always an option because of physical barriers that prevent movement, which are increasingly relevant for the many species influenced by the habitat fragmentation that results from anthropogenic land use change (Skole & Tucker, 1993; Smith et al., 2009). Unimpeded movement also requires adaptation to the novel habitat (Smith et al., 2009).

Adaptation in any form can be mediated by genes and/or by phenotypic plasticity (Sultan, 1995). Genetic adaptation happens through natural selection of beneficial alleles or genotypes, which are themselves introduced into a population by mutation, migration, or recombination (Carja, Liberman, & Feldman, 2014). By contrast, phenotypic plasticity, the ability for one genotype to produce different phenotypes when exposed to different environmental conditions (Stearns, 1989), is a within‐lifetime process (Charmantier et al., 2008), but not necessarily adaptive (e.g., Ghalambor, McKay, Carroll, & Reznick, 2007). The production of extreme phenotypes likely requires genetic adaptation (DeWitt, Sih, & Wilson, 1998). Although recent studies have shown that adaptive evolution can occur relatively quickly (Gingerich, 2009; Hendry, Farrugia, & Kinnison, 2008; Reznick, 2001), whether the rate of adaptation will be high enough to cope with environmental changes remains an open question (Chevin et al., 2010; Salamin et al., 2010).

Disentangling adaptive evolution and phenotypic plasticity is a critical component of understanding and predicting adaptive responses to environmental change, usually requiring common garden experiments conducted under controlled laboratory conditions (Moloney, Holzapfel, Tielbörger, Jeltsch, & Schurr, 2009). While these experiments are powerful, it is often difficult to translate the results into natural populations and extrapolate to naturally occurring phenotypic variation. One solution to this problem is provided by the many examples of invasive species that exhibit wide phenotypic variation and that reproduce asexually (e.g., Mergeay, Verschuren, & De Meester, 2006; Xie, Yu, Yu, & Liu, 2010). These taxa are powerful models for studying in situ adaptation through phenotypic plasticity, as by definition the environment they invade is novel (Sakai et al., 2001) and genotypic variation is held constant.

All of these criteria are met by *Potamopyrgus antipodarum*, a small ovoviviparous freshwater snail native to New Zealand. Populations of *P. antipodarum* in the native range are characterized by frequent coexistence of diploid sexual and polyploid (≥3x) asexual parthenogenetic lineages across a wide range of freshwater and brackish water habitats (Jokela, Lively, Dybdahl, & Fox, 1997; Neiman, Larkin, Thompson, & Wilton, 2012; Neiman, Paczesniak, Soper, Baldwin, & Hehman, 2011; Wallace, 1992). Since the mid‐19th century, *P. antipodarum* has successfully invaded Australia, Europe, the United States, Japan, and Chile (Alonso & Castro‐Díez, 2012; Bowler, 1991; Collado, 2014; Ponder, 1988; Shimada & Urabe, 2003); only asexual lineages are found out of the species' native range (Gangloff, 1998; Hauser, Carvalho, Hughes, & Carter, 1992; Hughes, 1996; Jacobsen, Forbes, & Skovgaard, 1996). Besides its status as a model for studying the maintenance of sex (e.g., Lively, 1987; Neiman & Lively, 2005), invasion biology (e.g., Alonso & Castro‐Díez, 2008; Dybdahl & Kane, 2005) and ecotoxicology (e.g., Gust et al., 2014; Matthiessen, 2008; Sieratowicz, Stange, Schulte‐Oehlmann, & Oehlmann, 2011; Völker, Gräf, Schneider, Oetken, & Oehlmann, 2014), *P. antipodarum* is notorious for its extreme variability in shell shape and size, especially—but not only—in its native range (Haase, 2008; Warwick, 1952; Winterbourn, 1970a). Because nearly all freshwater snails have a hard shell (Strong et al., 2008) that covers most or all of the body, and thus mediates environmental contact, it seems natural to assume that variation in shell size and shape is often adaptive. This assumption is premature, however, in light of the fact that we still lack a comprehensive understanding of the adaptive value of intraspecific morphological variation in this species. Accordingly, the asexual invasive populations of *P. antipodarum*, which exhibit pronounced variation in size and shape and occur in a variety of habitats, provide a powerful means of addressing questions regarding the role of phenotypic plasticity in morphological adaptation in natural populations.

Morphological variation in *P. antipodarum* has been at least tentatively linked to factors including water current, presence and absence of predators, parasitism, and depth. With respect to water current, Haase (2003) showed that New Zealand *P. antipodarum* living in environments with relatively rapid flow rates have larger and broader shells with a wider aperture and foot area relative to snails in environments with lower flow rate. A similar result was reported for invasive *P. antipodarum* and the native *Pyrgulopsis robusta* in the United States (Kistner & Dybdahl, 2014).

Spininess in native *P. antipodarum* appears to be, at least in part, a defense against predation: relatively large spines reduce predation pressure from common bullies (*Gobiomorphus cotidianus*). This advantage does not come cost‐free, however, because spines tend to collect seston, increasing the drag of the shell, with the consequence that large spines are likely less suitable for lotic habitats (Holomuzki & Biggs, 2006).

Native lake populations of *P. antipodarum* become wider, larger, and spinier with increasing depth (Jokela et al., 1997; Vergara, Fuentes, Stoy, & Lively, 2016). Whether this variation in size is linked to more intense parasitic pressure in shallower waters (Jokela, Dybdahl, & Lively, 1999; Jokela & Lively, 1995a, 1995b; Negovetic & Jokela, 2001) or to another factor related to depth (Vergara et al., 2016) remains unclear. Native *P. antipodarum* are intermediate hosts for at least 14 species of endoparasites including castrating trematodes (Hechinger, 2012; Winterbourn, 1974). Infection of juvenile *P. antipodarum* by parasites, and in particular castrating trematodes, may affect shell morphology and brood size through changes in resource allocation of the growing snail (Levri, Dillard, & Martin, 2005; Negovetic & Jokela, 2001). For example, infected *P. antipodarum* are wider and less spiny than uninfected counterparts (Lagrue, McEwan, Poulin, & Keeney, 2007; Levri et al., 2005). Infection by parasites of invasive *P. antipodarum* is, however, extremely rare: for instance, in Europe, only 28 snails of 5,788 (0.4%) collected in France were found to be infected (Gérard & Le Lannic, 2003), and only a single infected specimen has been reported in Poland (Zbikowski & Zbikowska, 2009). The apparent absence of parasite pressure in the introduced range is also a potential contributor to *P. antipodarum*'s success as an invader (Alonso & Castro‐Díez, 2012).

The size variation in *P. antipodarum* is especially notable because of the strong and positive relationship between size and the number of brooded embryos (McKenzie, Hall, & Guralnick, 2013), which is in turn likely at least in part caused by the likelihood that larger snails can harbor larger brood pouches.

To summarize the observations to date, shell morphology in both native and invasive *P. antipodarum* populations is highly variable and is likely to be at least partially linked to adaptation to environmental conditions. The vast majority of studies of shell morphology in *P. antipodarum* have used traditional length measurements to describe both size and shape. This type of approach does not allow for a clear separation of these two metrics, a limitation that can be overcome in the framework of geometric morphometrics (Bookstein, 1991; Zelditch, Swiderski, & Sheets, 2012). Recently, geometric morphometrics have been productively applied to investigate relationships between morphology and water current (Kistner & Dybdahl, 2013, 2014) and to compare lake populations (Vergara et al., 2016).

In this study, we used geometric morphometrics to evaluate the role of specific environmental factors in influencing variation in shell shape and size of invasive European *P. antipodarum* populations. Common garden experiments have shown that both genetic factors and phenotypic plasticity contribute to variation in *P. antipodarum* shell morphology (Kistner & Dybdahl, 2013). Here, we took advantage of the very low genetic diversity among European populations of *P. antipodarum* in an attempt to characterize the relative contribution of genetic variation and phenotypic plasticity to variation in shell morphology across 21 populations. Previous studies have suggested as few as four clones (nuclear microsatellites; Weetman, Hauser, & Carvalho, 2002) and two maternal lineages (mitochondrial 16S rRNA; Städler, Frye, Neiman, & Lively, 2005) in European *P. antipodarum*. We used cyt *b* and 16S rRNA mitochondrial markers (which also allowed us to relate our findings to previous analyses of Neiman & Lively, 2004; based on cyt *b* and Städler et al., 2005; who used 16S rRNA) and 50 single‐nucleotide polymorphism loci (SNPs; 16 established in Paczesniak, Jokela, Larkin, & Neiman, 2013 and 34 newly developed) to perform the first study of which we are aware to combine nuclear and mitochondrial markers to genotype invasive European *P. antipodarum* and provide a more in‐depth picture of genetic variation in these snails. We also used these markers to account for genetic relatedness and assess the contribution of phenotypic plasticity to the observed morphological variation. Usually, disentangling phenotypic plasticity and genetic adaptation requires manipulation in common garden or transplant experiments (Moloney et al., 2009), as already stated above. However, due to the low genetic diversity among invasive *P. antipodarum,* the distribution in Europe practically resembles a large, natural transplant experiment potentially allowing for this distinction as we will address in more detail in Discussion.

By evaluating the relationship of variation in shell morphology to environmental parameters, we aimed at establishing whether morphological variation might serve an adaptive function as a trait influencing the survival of snails in different habitats. In addition, we related the number of brooded embryos as a direct proxy of fitness to the same environmental parameters as well as to shell morphology in order to reveal potential interactions between the environment and morphology. In accordance with previous studies in *P. antipodarum*, we expected to find that both shell shape and size would vary with flow rate. By contrast, physicochemical parameters should largely affect metabolic processes and thus primarily influence size (see Gittenberger, Piel, & Groenenberg, 2004). Previous analyses have shown positive shell length–fecundity relationships in *P. antipodarum*, with longer females brooding more embryos (McKenzie et al., 2013). While it is difficult to predict the effect of shell shape on fecundity, we expected that broader snails would have larger distalt genitalia. Because this structure is where embryos are brooded, we thus predicted that there would be a positive relationship between shell breadth and embryo number (also see McKenzie et al., 2013).

## 2 MATERIALS AND METHODS

### 2.1 Collection

Snails were collected at 22 sites across Belgium (eight sites), Germany (12), and the Netherlands (two) during the summers of 2015 and 2016 (Table 1). All snails were collected during daylight with a small dip net, which we used to scrape individual snails off the lake/stream bottom or hard surfaces and aquatic plants at depths between 0 and 50 cm. We collected snails at a depth of 2 m by scuba diving at one location (DEJAT). Samples DEHOT and DEHOB are from the same location but were sampled 1 year apart. Snails were fixed upon collection in 96% ethanol. During the time of collection, we also recorded water temperature, salinity, conductivity, pH, concentration of nitrites and nitrates, flow rate (none, low, and high), coverage of the site against sunlight (%), and water turbidity (clear and unclear). Because these water bodies are not continuously monitored for these parameters, we had to assume that our single measurements reflect general differences between sites.

**Table 1:** Sampling sites

Acronym	Country	Location	Date	Coordinates	Altitude (m)	*N*
BEBRA	Belgium	Brakel, Oost‐vlaanderen	August 2015	N 50°45′50.9″, E 3°47′37.7″	38	18
BEGER		Geraardsbergen, Oost‐vlaanderen	August 2015	N 50°49′06.3″, E 3°54′07.8″	23	18
BEHER		Kleine Nete, Herentals, Antwerpen	July 2015	N 51°11′11.0″, E 4°49′55.0″	10	10
BEKAS		Affluent of Kleine Nete, Kasterlee, Antwerpen	July 2015	N 51°13′41.8″, E 4°58′43.0″	17	7
BEOOE		Oostkamp, West‐vlaanderen	July 2015	N 51°8′41.8″, E 3°16′13.5″	6	18
BEOOT		Oostkamp, West‐vlaanderen	July 2015	N 51°7′55.6″, E 3°16′15.6″	7	20
BEVEU		Veurne, West‐vlaanderen	July 2015	N 51°0′52.2″, E 2°34′44.4″	−2	20
BEWIL		Wilskerke, West‐vlaanderen	August 2015	N 51°11′22.3″, E 2°51′34.7″	0	20
DEBIN	Germany	Binnenwasser, Neustadt, Schleswig‐Holstein	September 2015	N 54°6′28.6″, E 10°48′36.6″	1	20
DEDOB		Dobersdorfer See, Dobersdorf, Schleswig‐Holstein	September 2015	N 54°19′51.8″, E 10°17′4.3″	29	20
DEHOB		Mühlbach, Hohen Sprenz, Mecklenburg‐Vorpommern	July 2016	N 53°55′24.2″, E 12°11′57.7″	27	20
DEHOT		Mühlbach, Hohen Sprenz, Mecklenburg‐Vorpommern	July 2015	N 53°55′24.2″, E 12°11′57.7″	27	16
DEJAR		Kiessee, Jarmen, Mecklenburg‐Vorpommern	July 2016	N 53°55′44.5″, E 13°18′60.0″	5	20
DEJAT		Kiessee (**2 m deep**), Jarmen, Mecklenburg‐Vorpommern	July 2016	N 53°55′45.3″, E 13°18′58.5″	5	20
DEPAS		Passader See, Passade, Schleswig‐Holstein	September 2015	N 54°21′51.7″, E 10°18′56.4″	19	20
DERUG		Quellsumpf Ziegensteine, Klein Stresow, Rügen, Mecklenburg‐Vorpommern	July 2015	N 54°21′23.7″, E 13°36′27.0″	19	16
DESEG		Lake north of Südsee, Gießen, Hessen	September 2016	N 50°34′4.08″, E 8°37′39.7″	154	20
DESEL		Selender See, Pülsen, Schleswig‐Holstein	September 2015	N 54°19′17.9″, E 10°27′7.5″	37	20
DEWEL		Westensee, Wrohe, Schleswig‐Holstein	September 2015	N 54°16′8.4″, E 9°57′39.9″	7	20
DEWER		Westensee river, Wrohe, Schleswig‐Holstein	September 2015	N 54°16′39.2″, E 9°54′5.8″	8	20
DEWIT		GroßWittensee, Schleswig‐Holstein	September 2015	N 54°24′6.7″, E 9°46′11.5″	7	19
NL1	Netherlands	Valkenburgse Meer, Katwijk, South Holland	June 2016	N 52°09′25.2″, E 4°26′31.2″	−2	19
NL2		Katwijk aan Zee, South Holland	June 2016	N 52°12′34.6″, E 4°24′9.9″	4	15

### 2.2 Determinate growth experiment

Prior to shape and size analyses, we had to ensure to the extent possible that all individuals belonged to the same developmental stage. Previous studies in *P. antipodarum* have typically used a shell length or whorl number threshold to indicate sexual maturity, that is, the stage when first embryos are produced. This threshold was usually equated with adulthood, although it is often explicitly acknowledged that *P. antipodarum* can sustain growth after releasing their first offspring. In addition, growth estimates from individual studies are often assumed to represent the entire species. This assumption poses a problem in light of the wide morphological variation that characterizes *P. antipodarum*; for example, the shell height of fully grown snails varies across populations by a factor of about 2.5 (unpublished data). Among taxonomists, it is well established that many groups of gastropods, including the Tateidae family to which *P. antipodarum* belongs, have determinate growth, with the terminus indicated by a thickened, continuous apertural lip (e.g., Haase, 2003). Even so, this assumption has never been explicitly tested in many species, including *P. antipodarum*. Instead, this determinate growth assumption is implicitly supported by the restricted variance (usually < 10%) of length measurements within populations (e.g., Haase, 2008). We here used shell height measurements for living individuals to confirm experimentally that *P. antipodarum* stops growing once the lip is continuous. In other words, we considered a snail to be adult and fully grown once the apertural lip is completed. All snails subsequently analyzed fulfilled this criterion. By defining a clear criterion for adulthood, we thus provided an unambiguous basis for statistical comparisons.

**Figure 1:**
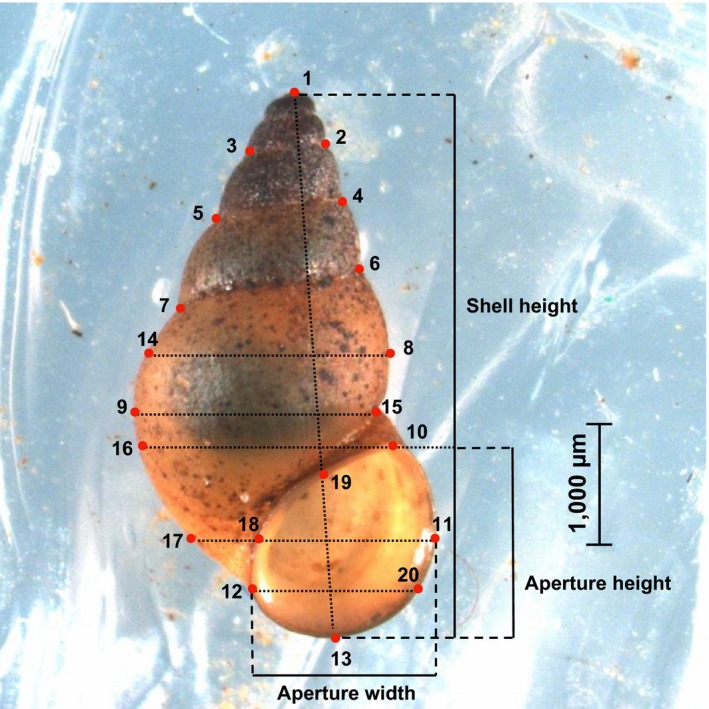
Landmarks (LM) used in geometric morphometric analyses and measurements: apex (LM1); intersection of sutures with the shell outline (LMs 2–7); most external right (LM8) and left (LM9) points of the body whorl; highest (LM10), lowest (LM13), most external left (LM12), and right (LM11) point of the aperture; dotted auxiliary lines indicate how landmarks 14–20 were placed

To test this assumption of determinate growth, we collected snails from three different sites: DEHOT and DEJAT (Table 1), and from a small New Zealand stream in the Buller Gorge of the South Island (S 41°50′6.695″; E 171°40′0.181″). Prior to the initiation of the experiment, we housed the snails in aquariums (29 × 14 × 16 cm) filled with 4L of artificial pond water (APW, 0.5 g/L sea salt, Tropic Marine^®^, Germany; Symanowski & Hildebrandt, 2010), placed in an 18°C climate cabinet with 16:8 hr day–night regime. Weekly, we added deionized water to compensate for evaporation and fed the snails with *Spirulina* (40%)‐based staple food (JBL, Germany), which also includes cereals, vegetables, fish byproducts, mollusks, and crustaceans. Once a month, half of the water was changed in each aquarium. In July 2016, seven snails from each population with a completely formed aperture lip were isolated in glass cups (8 cm diameter) containing 200 ml of APW and a clean granite stone. The cups were placed in the same climate cabinet with same temperature and light conditions as the snails had been previously housed. Weekly, we added deionized water to compensate for the evaporated water and then changed 100 ml of water with a syringe. The snails were fed with *Spirulina* flakes, and cup placement was arbitrarily rotated in the cabinet once a week. The snails were photographed and shell height (Figure 1) measured twice within a 10‐month interval under a Nikon stereo SMZ18 microscope with a 0.5x SHR Plan Apo lens at 1.5× magnification; each snail was first placed on a wet towel with the aperture facing up and the coiling axis oriented horizontally. After the photograph was taken, we then placed each snail back in its respective cup. We used a *t* test to compare shell height measurements in the first and the last month.

### 2.3 Morphometrics

Up to 20 (when at least 20 snails were found, otherwise the maximum sampled) shells from fixed adult snails per location (Table 1) positioned as described above were photographed under a Carl Zeiss Discovery V20 microscope with an AxioCam MRc camera and a Plan Apo S 0.63x objective at 1.5× magnification. We then used the Axio Vision microscope software (Zeiss) to measure shell height and aperture height and width (Figure 1). We classified shells as either smooth or ridged; no spiny shells were observed (Holomuzki & Biggs, 2006). We counted the number of whorls to the nearest eighth of a whorl (Haase, 2003). Because the number of whorls was correlated with shell height (Spearman's correlation, *r*s* *= .674, *p *<* *.001), we dropped whorl number from subsequent analyses including shell height.

### 2.4 Geometric morphometrics

We used the framework of geometric morphometrics to analyze shell shape and size independently (Bookstein, 1991; Zelditch et al., 2012). After transforming shell images into Thin‐Plate Spline (tps) format, we placed 20 Cartesian landmarks (Figure 1) with the programs tpsUtil version 1.64 (Rohlf, 2012) and tpsDig version 2.22 (Rohlf, 2010), respectively. A Procrustes superimposition of the landmarks, which filters out the location, scale, and rotational information of the landmarks (Rohlf & Slice, 1990), and centroid size (CS) were calculated with both CoordGen8 (Sheets, 2011) and MorphoJ v.1.06 (Klingenberg, 2011). CS is calculated as the square root of the summed squared distances of each landmark from the centroid of the landmark configuration (Bookstein, 1991). This CS value represents the overall size of the shell and is the only size measurement independent of shape. We used a repeatability test (Goodall's *F* test, *F *=* *0.390, *df* = 36, 1296; *p *=* *.999) implemented in the TwoGroup8 software (Sheets, 2011) for 20 snails photographed twice with an interval of 1 month to demonstrate that snails were photographed and landmarks digitized in a consistent manner. This indicated that any detected variance in shape would be unrelated to manipulative error (Schilthuizen & Haase, 2010). Because a multiple linear regression of Procrustes coordinates on CS (Monteiro, 1999) indicated allometry (*R* ^2^ = .031; mean squared error <0.001; *p *<* *.001), we used the regression residuals in our further analyses, thereby removing the portion of variance of shape explained by size (Klingenberg, 2016).

### 2.5 Dissections

Shells were gently dissolved in 0.5 mol/L EDTA (pH 7.5) for 2 days. We then dissected the now‐exposed soft body under a microscope, determined sex (presence of a penis for males), and counted the number of embryos brooded by each female. Any embryos were then removed and the head of each snail placed back into 96% ethanol to be sent to LGC Genomics (http://www.lgcgroup.com) for DNA extraction, mtDNA sequencing, and SNP genotyping. We assumed that embryo counts were comparable across samples because all snails were collected during the summer months, the presumptive reproductive peak (McKenzie et al., 2013; Schreiber, Glaister, Quinn, & Lake, 1998). Because infection status (and male vs. female status) can affect shell shape and size in *P. antipodarum* (e.g., Lagrue et al., 2007; Levri et al., 2005), we also determined whether macroparasites were present in the body cavity.

### 2.6 Genetic analyses

DNA was extracted with the sbeadex^™^ lifestock kit in conjunction with an RNase treatment. A 481‐bp fragment of the mitochondrial 16S ribosomal RNA gene (16S; primers S1‐Universal (5′‐CGGCCGCCTGTTTATCAAAAACAT‐3′) and S2‐Potamo (5′‐GTGGTCGAACAGACCAACCC‐3′; Städler et al., 2005)) and a 497‐bp fragment of the mitochondrial cytochrome *b* gene (cyt *b*; primers 5′‐TTCTTTATTAGGACTTTGTTTAGG‐3′ and 5′‐TTTCACCGTCTCTGTTTAGCC‐3′; Neiman & Lively, 2004) were sequenced on an Illumina MiSeq V3 platform and clustered with CD‐HIT‐EST v 4.6.1 (http://weizhonglab.ucsd.edu/cd-hit/). A nucleotide BLAST was performed to compare our sequences to the NCBI database (https://blast.ncbi.nlm.nih.gov/Blast.cgi). Haplotype distributions were mapped with PopART (http://popart.otago.ac.nz/index.shtml).

We also used SNP loci to characterize the clonal identity of our snails. Twenty‐three of these SNPs were already available (https://www.ncbi.nlm.nih.gov/projects/SNP/snp_viewBatch.cgi?sbid=1059300; Paczesniak et al., 2013), and 39 SNPs were newly developed. For the latter, we mapped RNA‐Seq reads obtained from the NCBI Sequence Read Archive (SRA) of a female *P. antipodarum* individual from New Zealand lake Alexandria (SRA accession: SRS2839272) against 3200 randomly selected contigs from the *P. antipodarum* transcriptome assembly (Bankers et al., 2017; DDBJ/EMBL/GenBank accession: GFLZ00000000) with Bowtie2 (Langmead & Salzberg, 2013). We then used SAMtools MPileup version 2.0 (Li et al., 2009) to call single‐nucleotide variants of the mapped reads. We selected candidate neutral SNPs (3rd codon position) that were flanked by 50 bp of invariable sequences both upstream and downstream, which were required for primer design. The reading frames of the contigs were determined with ORFfinder (https://www.ncbi.nlm.nih.gov/orffinder/). Only one candidate SNP per contig was selected. The sequences of the resulting 90 candidate SNPs were sent to LGC to design KASP^™^ assays based on the primer design parameters calculated by their Kraken^™^ software (https://www.lgcgroup.com/products/genotyping-software/kraken/#.WW8t3WhLe70).

Validation of the KASP assays was performed on 22 specimens from each of three New Zealand populations collected in February and March 2016. Population one was collected from a small stream flowing into the Waitawheta River (S 37°27′47.087″; E 175°46′48.068″) located in the Waikato region of the North Island, population two was collected from the Waitawheta river itself (S 37°27′57.386″; E 175°46′50.453″), and population three (S 41°14′59.234″; E 172°10′59.697″) was collected from the catchment of the Karamea River in the northwest of the South Island. We used native populations of *P. antipodarum* for the validation because of their higher genetic diversity and the potential presence of both sexual diploid and asexual polyploid lineages (Dybdahl & Lively, 1995; Neiman & Lively, 2004; Paczesniak et al., 2013; Städler et al., 2005). The snails were dissected prior to DNA extraction, sexed to infer whether a population was reproducing sexually or clonally, and checked for parasites in order to avoid contamination. Populations two and three had both females and males, while all dissected snails in population one were females. None of the dissected snails were infected with parasites.

Of the 90 candidate SNPs, 64 loci fulfilled the LGC quality criteria. We selected 39 loci of the 64 loci (European Variation Archive accession number: PRJEB24869) that were scorable in >85% of the individuals. 21 of these loci were polymorphic in the validation and 18 were fixed in the three New Zealand populations. Thus, a total of 62 loci were available for genotyping 425 European *P. antipodarum* (Table S1).

Most of the SNP loci were heterozygous (77% of polymorphic loci) in at least some snails. For phylogenetic analyses of nuclear data, the polymorphisms have either to be phased, that is, attributed to haplotypes, or recoded with IUPAC ambiguity codes, which would unduly reduce the information content. However, because European *P. antipodarum* reproduce by apomictic parthenogenesis (i.e., without recombination; Winterbourn, 1970b), we could safely ignore phases and use the genotypic information as follows: KASP can detect polymorphisms but does not allow the distinction of allele dosage (e.g., AAG vs. AGG). Accordingly, we concatenated all variable SNP loci with two positions, one for each allelic state in alphabetical order such that a homozygous locus was represented by two identical states and a heterozygous locus by different states in our alignment. This alignment was used to reconstruct a median‐joining network using PopART.

### 2.7 Statistical analyses

The variation in shape among individuals and populations, represented by the regression residuals of Procrustes coordinates on CS, was first visualized using principal component (PCA) and canonical variate analyses (CVA) and wireframe graphs. Pairwise population comparisons were based on Procrustes distances, a measure of the absolute magnitude of the shape deviation (Klingenberg & Monteiro, 2005), tested with 10,000 permutation rounds. The variation in aperture size (likely correlated with foot size; Haase, 2003) relative to the shell height was tested against the PC1 shape axis with a correlation; aperture size was approximated as the area of an ellipse using aperture height and width as the ellipse axes.

We used a linear mixed model (LMM) to describe the effects of the environmental factors on shape, using PC1 as response variable, and by setting the mitochondrial haplotypes and the SNP genotypes (nested in haplotypes) as random factors to control for variation of shape due to genetic lineage. A marginal *R* ^2^, representing the proportion of variance explained by the fixed variables alone, and a conditional *R* ^2^, representing the proportion of variance explained by both the fixed and random factors (Vonesh, Chinchilli, & Pu, 1996), were calculated using the r.squared GLMM function (Johnson, 2014; Nakagawa & Schielzeth, 2013) of the *MuMIn* v.1.40.0 package (Bartoń, 2017). Salinity and longitude were removed from the environmental variables because they were positively correlated with conductivity (Kendall's *Tau* = 0.933, *z* = 26.544, *p *<* *.001) and latitude (*Tau* = 0.38, *z* = 11.051, *p *<* *.001), respectively, and flow rate and water transparency were treated as ordinal variables. Before running the LMM, continuous variables were rescaled with the lapply (Becker, Chambers, & Wilks, 1988) R function. The same environmental variables and random genetic factors were also used in all generalized linear mixed models (GLMMs) analyzed in this study and described below. The potential influence of flow rate on the relative aperture area was tested with a Welch *F* test. To provide further insight into whether relative aperture area might reflect adaptation to flow, we evaluated the relationship of this morphological variable to the number of brooded embryos normalized by size (embryos/CS) in three independent correlations for habitats with no, low, and high flow. We used a GLMM to evaluate the relationship of shell smoothness (smooth or ridged) against the environmental variables.

Variation in shell size (CS) was visualized with box plots across populations and evaluated using a GLMM with environmental data as explanatory variables. We evaluated variation in CS between DEJAR and DEJAT and between DEHOB and DEHOT with *t* tests. A final GLMM with number of embryos as response variable as a proxy for fitness was used to test the direct influence of shape (PC1), CS, the environmental variables, and the interaction of PC1 and CS with the environmental variables on fecundity. Finally, we used correlations to address the relationship between the number of brooded embryos and size and shape within each population.

All statistical tests were executed in PAST v.3.14 (Hammer, Harper, & Ryan, 2001), MorphoJ, or R v.3.3.3 (R Development Core Team, 2011). The LMM and GLMMs were run with the *lme4* v.1.1‐13 package (Bates, Maechler, Bolker, & Walker, 2015) and built by dropping terms based on type‐II Wald Chi‐square tests of the Anova function available from the *car* package (Fox & Weisberg, 2011). Positive or negative effects of factors were visualized with plots using the *effects* v.3.2 package (Fox, 2003). Nonparametric tests were used if normal distributions were rejected with a Shapiro–Wilk test, and we adjusted the significance level in table‐wide comparisons with the Bonferroni correction.

## 3 RESULTS

### 3.1 Mitochondrial haplotypes and nuclear genotypes identified in sample sites

We detected only two different mitochondrial haplotypes for 16S and cyt *b* in the 425 genotyped snails. One of the two 16S haplotypes was an exact match (BLAST: 100% query cover and identity) with the previously discovered European haplotype t (Städler et al., 2005). The second 16S sequence differed by one mutation (100% query cover, 99% identity, G instead of T at site 456) from the previously established European haplotype *z* (Städler et al., 2005); we here refer to this second haplotype as z2 (GenBank accession number: MG581815). Haplotypes t and z2 differ from each other at six of 481 sites (1.25%). Our most common cyt *b* sequence (92% of samples) was an exact match with the previously established New Zealand haplotype 22, and the second cyt *b* haplotype (8% of samples) was an exact match with the previously described haplotype 37 (Neiman & Lively, 2004; Neiman et al., 2011). Haplotypes 22 and 37 also differ from each other by six mutations (1.21%). In all cases, haplotype t and haplotype 22 and haplotype z2 and 37, respectively, were found in the same individuals. We will henceforth refer to these haplotype pairs as haplotypes t/22 and z2/37.

**Figure 2:**
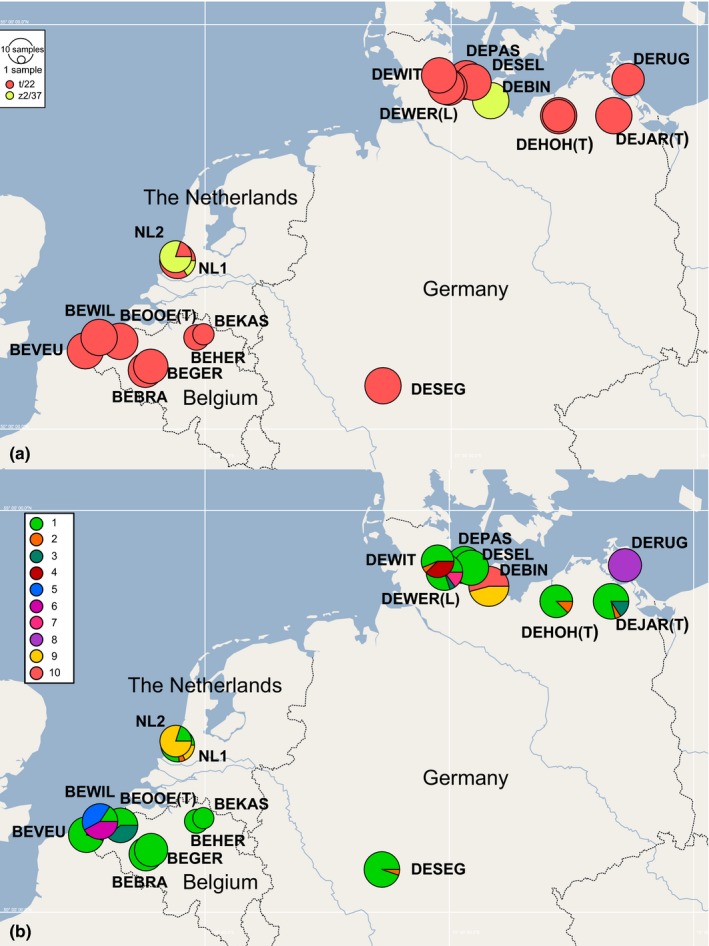
Northwest European distribution of the mtDNA (16S/cyt b) haplotypes (a) and the SNP genotypes (b). Size of circles proportional to number of sequenced individuals (up to 20 per site)

**Figure 3:**
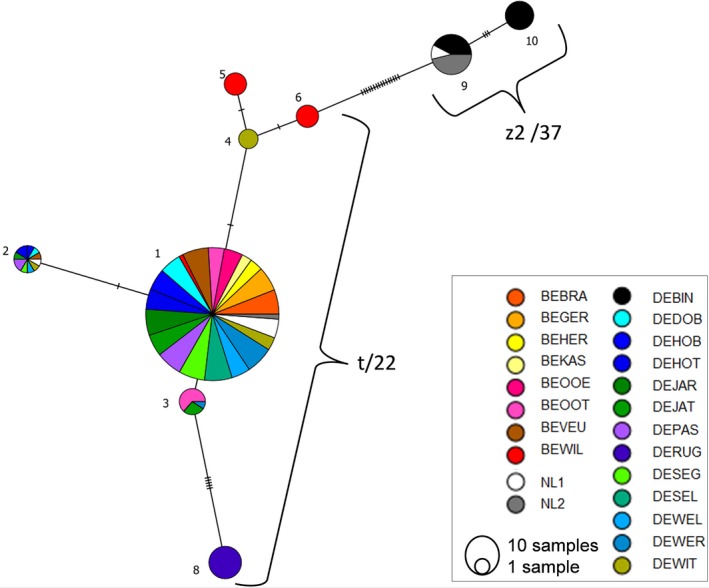
Median‐joining network for the 10 SNP genotypes found in 425 European individuals of *Potamopyrgus antipodarum*. Colors represent sampling locations, each branch a single‐nucleotide substitution, and short transversal lines unsampled genotypes. Size of circles and segments proportional to number of individuals per genotype and location, respectively. SNP genotype 7 is not plotted as it was only found in four individuals

Haplotype t/22 was the dominant lineage, present in 390/425 snails (92%) and at 20 of our 21 localities (95%). Only 35 snails (8%) from three sites, including our only brackish site DEBIN and both sites from the Netherlands (NL1 and NL2), had haplotype z2/37 (Figures 2a and 3).

Of the 62 SNP loci, 50 could be consistently genotyped across all samples. Thirty‐four of the 50 SNPs (68%) were fixed across all snails and were thus not used for further analysis. The other 16 SNPs (32%) were polymorphic and combined to produce ten genotypes (Figure 2b, Table 2). Eight of these genotypes had mtDNA haplotype t/22, and the other two had haplotype z2/37 (Figure 3, Table 2).

**Table 2:** SNP genotypes. Nucleotides that were identical to the first sequence were replaced by a dot; ?, missing information

SNP genotype	mtDNA haplotype	comp140766_c3_seq1		comp141987_c0_seq1		comp144295_c3_seq1		comp152737_c0_seq2		comp152973_c0_seq1		comp162701_c0_seq4		comp163630_c0_seq1		comp132525_c0_seq1		comp141103_c0_seq2		comp146583_c0_seq2		ss804270605		ss804270596		ss804270598		ss804270584		ss804270595		ss804270614	
		A	B	A	B	A	B	A	B	A	B	A	B	A	B	A	B	A	B	A	B	A	B	A	B	A	B	A	B	A	B	A	B
1	t/22	C	C	T	C	T	A	A	A	G	G	T	C	T	C	C	A	T	T	G	G	G	A	A	A	C	C	T	C	T	C	T	C
2		.	.	.	.	.	.	.	.	.	.	.	.	.	.	.	.	.	.	.	.	.	G	.	.	.	.	.	.	.	.	.	.
3		.	.	.	T	.	T	.	.	.	.	.	.	.	.	.	.	.	.	.	.	.	.	.	.	.	.	.	.	.	.	.	.
4		.	.	.	.	.	.	.	.	.	.	.	.	.	.	.	.	.	.	.	.	.	.	.	.	.	.	C	.	.	.	.	.
5		.	.	.	.	.	.	.	.	.	.	.	T	.	.	.	.	.	.	.	.	.	.	.	.	.	.	C	.	.	.	.	.
6		.	.	.	.	.	.	G	.	.	.	.	.	.	.	.	.	.	.	.	.	.	.	.	.	.	.	C	.	.	.	.	.
7		.	.	.	.	.	T	.	.	.	.	.	.	.	.	.	.	.	.	.	.	.	.	.	.	.	.	.	.	.	.	.	T
8		.	.	.	T	?	?	.	.	.	.	.	T	C	.	.	C	.	.	.	.	.	.	.	.	.	.	.	T	.	T	?	?
9	z2/37	T	T	C	.	A	.	G	G	.	A	C	.	.	T	A	.	C	C	.	C	A	.	G	.	T	T	C	.	C	.	.	T
10		T	T	C	.	?	?	G	G	A	A	C	.	.	T	A	.	C	C	C	C	A	.	G	G	T	T	C	.	C	.	?	?

### 3.2 *Potamopyrgus antipodarum* exhibits determinate growth

**Figure 4:**
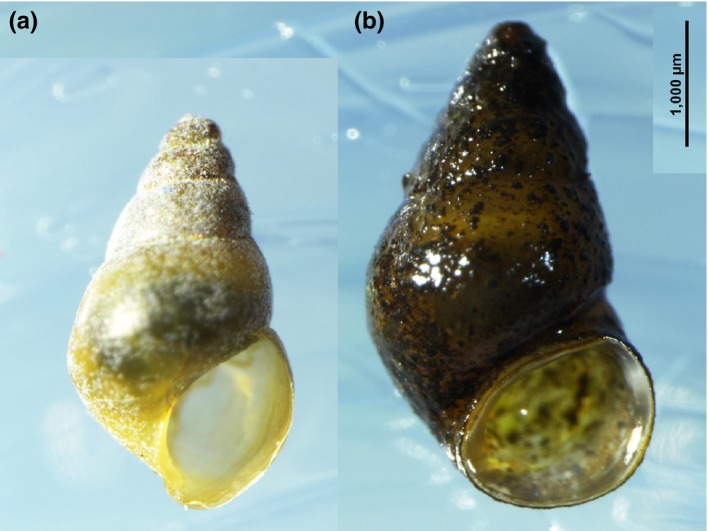
Comparison of juvenile (a) and adult (b) *Potamopyrgus antipodarum*. Adults are identified by the thickened continuous apertural lip

Our growth experiment confirmed that snails deemed to be adult by the criterion of a continuous and thickened apertural lip did not grow over the following 10 months (*t *=* *−0.463, *p *=* *.647), indicating that *P. antipodarum* has determinate growth (Figure 4).

### 3.3 Sex and parasitism unlikely to influence shell morphology measurements

All dissected snails were females and none were parasitized, meaning that we did not have to control for or otherwise address an effect of sex or infection on shell morphology. The number of embryos per female varied between 0 and 90 (median = 23).

### 3.4 Shell shape is most strongly influenced by genetic factors

**Figure 5:**
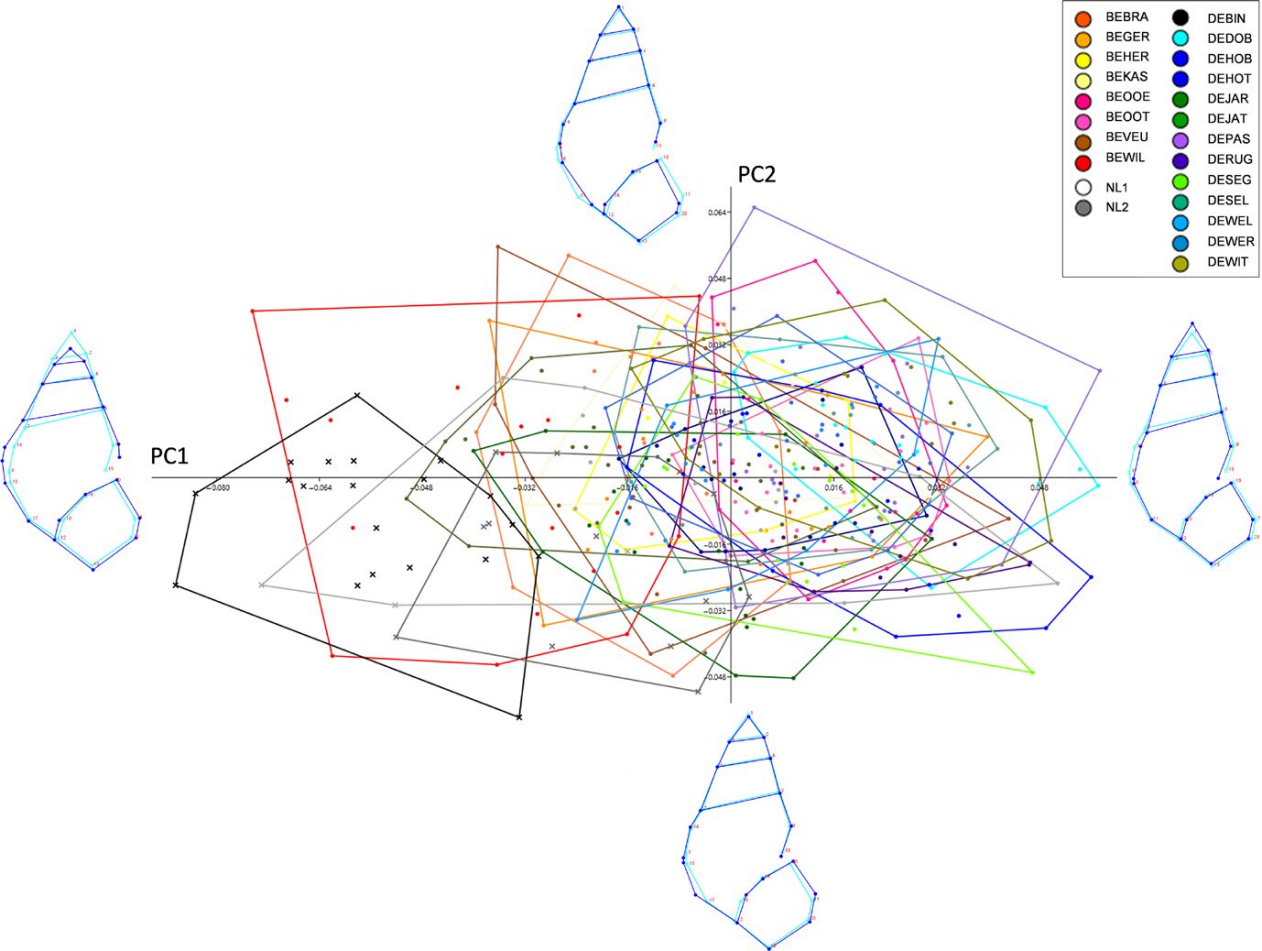
Principal component analysis illustrating variation in shell shape. Convex hulls represent different sampling locations, crosses snails with haplotype z2/37, and dots haplotype t/22. The wireframes show the variation (exaggerated 10 times) in shape (dark blue) against the mean shape (light blue) along the PC axes

Variation in shape across all samples is illustrated by the PCA in Figure 5 and among populations by the CVA in Figure S1. Principal components (PC) 1–3 explained 55.68% of the total shape variance and axes 1–3 (CV) of the CVA 58.38%. The wireframe graphs inserted into these plots illustrate the morphological transformations along the axes: for example, shorter but wider shells at negative PC1 and CV1 values to longer but narrower snails at positive PC1 and CV1 values. Pairwise Procrustes distances across populations are given in the Table S2. Most notably, DEBIN was different from all other samples, NL2 was different from all other samples except NL1, and BEWIL differed from all other samples except BEKAS. There was no difference in shape between DEJAR and DEJAT (same location, different depth) and DEHOH and DEHOT (same location, different sampling years). Within haplotype t/22, the relative aperture area was negatively correlated with PC1 (Spearman's *r*s* *= −.387, *p *<* *.001): that is, the wider the shell, the larger the aperture (also see the wireframes accompanying the PCA (Figure 5)).

**Figure 6:**
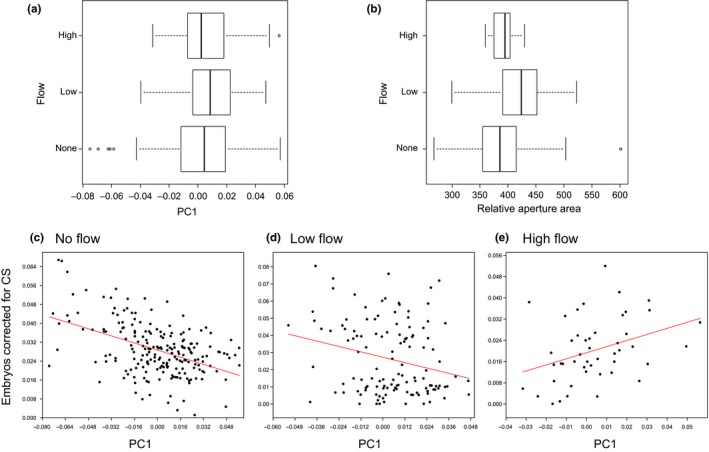
Relationships of shape, brood size, and flow. Box plots for PC1 (a) and relative aperture area (b) for three classes of flow rate for haplotype t/22. Correlation plots for number of embryos corrected for size (CS) relative to shape (PC1) for no (c), low(d), and high‐flow rate (e). Shells with high PC1 values have a slender shape compared to those with lower PC1 values

The LMM with shape (PC1) as response variable showed that the random factors haplotype and genotype and the fixed factors temperature, latitude, and nitrate had a significant effect. There was however no evidence for a significant effect of flow rate (Figure 6a), conductivity, pH, nitrite, turbidity, sunlight coverage, or sampling month (Table 3). The variance explained by both random and fixed factors was 88.4% (= conditional *R* ^2^), almost all of which explained by the genetic factors (86.3%), leaving only 2.1% of the variance (= marginal *R* ^2^) associated with the fixed factors. Among the random factors, haplotype explained 10 times more variance than genotype, and among the fixed factors, temperature had a negative effect (inducing a wider shell), and latitude and nitrate a positive effect (inducing a slender shell) on shell shape.

**Table 3:** (a) Coefficients of random factors in the mixed models with shell shape (PC1), centroid size (CS), smoothness, and embryos as response variables, respectively. (b) Coefficients of fixed factors in the mixed models. Coefficients for the categorical factors (flow and turbidity) are related to the following categories, respectively: no flow, clear

(a)	Groups	Variance	*SD*
Shape	Haplotype (genotype) intercept	0.0003	0.0161
	Haplotype intercept	0.0023	0.0485
	Residual	0.0004	0.0187
CS	Haplotype (genotype) intercept	2.382e‐09	4.881e‐05
	Haplotype intercept	0	0
	Residual	0.0033	0.0577
Smoothness	Haplotype (genotype) intercept	0	0
	Haplotype intercept	4.4450	2.1080
Embryos	Haplotype (genotype) intercept	0	0
	Haplotype intercept	6.511e‐19	8.069e‐10

(b)		Estimate	*SE*	*t*	*p*
Shape (Gaussian family)	Intercept	−0.0308	0.0349	−0.883	—
	Latitude	0.0049	0.0010	4.445	—
	Temperature	−0.0051	0.0013	−3.879	—
	Nitrate	−0.0036	0.0017	−2.082	—
CS (Gamma family)	Intercept	0.0012	4.247e‐05	27.795	<.0001
	Flow (low)	−0.0002	9.024e‐06	−22.889	<.0001
	Flow (high)	−0.0002	1.007e‐05	−18.558	<.0001
	Nitrate	2.231e‐05	6.963e‐06	3.204	<.0001
	Latitude	−3.008e‐05	5.051e‐06	−5.955	<.0001
	Coverage	3.538e‐05	5.172e‐06	6.841	<.0001
	Turbidity	−5.168e‐05	8.327e‐06	−6.206	<.0001

		Estimate	*SE*	*z*	*p*
Smoothness (Binomial family)	Intercept	−1.9378	1.5303	−1.266	.2054
	Turbidity	−1.3773	0.6777	−2.032	.0421
Embryos (Poisson family)	Intercept	3.5802	0.0296	121.06	<.0001
	CS	0.4252	0.0342	12.450	<.0001
	PC1	−0.1584	0.0274	−5.780	<.0001
	Month	0.1854	0.0218	8.500	<.0001
	Conductivity	0.0425	0.0120	3.550	.0004
	Latitude	−0.0986	0.0178	−5.540	<.0001
	Flow (low)	−0.6776	0.0485	−13.980	<.0001
	Flow (high)	−0.6490	0.0727	−8.930	<.0001
	Turbidity	−0.2177	0.0326	−6.670	<.0001
	pH	−0.0956	0.0222	−4.300	<.0001
	Nitrite	−0.1096	0.0155	−7.060	<.0001
	CS × flow (low)	0.1375	0.0397	3.460	.0005
	CS × flow (high)	−0.0900	0.0546	−1.650	.0994
	CS × turbidity	−0.2095	0.0253	−8.290	<.0001
	PC1 × flow (low)	0.0394	0.0633	0.620	.5340
	PC1 × flow (high)	0.6870	0.0626	10.980	<.0001
	PC1 × pH	0.0896	0.0323	2.770	.0056
	PC1 × nitrate	0.1612	0.0250	6.440	<.0001
	PC1 × coverage	−0.1650	0.0194	−8.490	<.0001

*SD*, standard deviation; *SE*, standard error.

Shape alone and in interaction with flow rate, nitrate, sunlight coverage, and pH had an influence on fecundity (Table 3), with higher embryo production in snails with relatively wide versus slender shells. Even so, snails with slender shells had relatively high embryo number when compared to wider‐shelled snails in environments with high flow (Figure 6c–e), high nitrate concentration, high pH, or low sunlight coverage. The implications are that shape was associated directly with fecundity and indirectly via interaction with several environmental factors. Snails living in lotic habitats had a larger aperture area relative to their body height compared to snails from lentic habitats (Welch's *F *=* *22.43, *df* = 80.01, *p *<* *.001, Mann–Whitney pairwise *U* tests, no vs. low flow: *U *=* *6876, *p *<* *.001; no vs. high flow: *U *=* *2,100, *p *=* *.001; low vs. high flow: *U *=* *1,840, *p *=* *.949; Figure 6b). The number of embryos corrected for CS was positively correlated with the relative aperture area in both habitats with no flow (Spearman's *r*s = .201, *p *=* *.004) and low flow (Spearman's *r*s = .628, *p *<* *.001), but not with high flow (Spearman's *r*s = .258, *p *=* *.141). The slope of the embryo–CS relationship was three times steeper for low flow versus no flow.

Only 5% of the snails had a ridged shell. The presence/absence of ridges was associated with haplotype but not genotype, and only with turbidity among the fixed factors. Most (15 individuals, 75%) ridged snails had haplotype z2/37 and lived in clear waters. Our results suggest a strong genetic component for this character (Table 3).

### 3.5 Size is most strongly influenced by environmental factors

**Figure 7:**
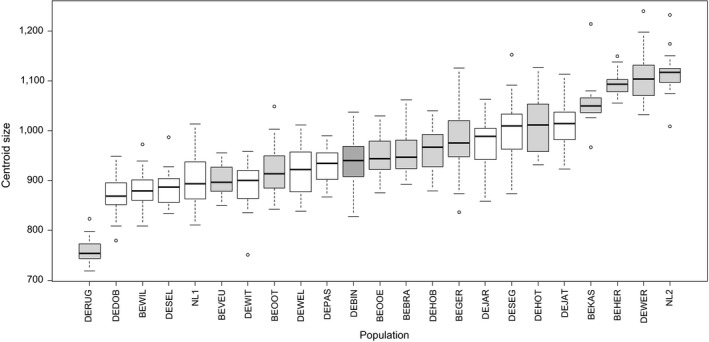
Size variation across populations. White = lake sites, light gray = river sites, and dark gray = brackish water site. For population abbreviations see Table 1

Size measured as CS and shell height varied by a factor of 1.5 across samples, with DERUG (CS = 760.63 ± 26.87; height = 3.48 ± 0.13 mm—mean ± *SD*) and NL2 (CS = 1117.29 ± 48.38; height = 5.00 ± 0.32 mm) having the smallest and largest shells, respectively (Figure 7). Only environmental factors had an effect on CS, with flow rate, latitude, and turbidity positively influencing CS, and nitrates and sunlight coverage negatively associated with CS (Table 3). Among these factors, only the interactions of size with flow rate and turbidity, respectively, had an effect on the number of embryos. Generally speaking, larger snails brooded more embryos, although the relationship between size and embryo number was weaker as flow rate and water turbidity increased.

Snails from deeper water in Jarmen (DEJAT; CS = 1011.79 ± 48.42; height = 4.54 ± 0.24 mm) were significantly larger than their counterparts collected from shallow water (DEJAR; 973.56 ± 48.42; 4.37 ± 0.23 mm; *t* test, *t *=* *−2.496, *p *=* *.017; *t *=* *−2.237, *p *=* *.031). Snails collected in 2015 from DEHOT (1010.21 ± 54.80; 4.61 ± 0.29 mm) were significantly larger and higher than snails collected in 2016 from the same site (DEHOB; 962.78 ± 47.12; 4.42 ± 0.25 mm; Mann–Whitney, *U *=* *86, *z* = −2.339, *p *=* *.019; *t *=* *1.679, *p *=* *.010).

### 3.6 Environmental factors and not genetic affiliation directly influence brood size

There was no apparent effect of genetic affiliation on the number of brooded embryos (Table 3). Besides the interactions with shape (PC1) and size (CS) mentioned above, a number of environmental factors directly influenced brood size. In particular, conductivity and sampling month had a positive effect and latitude, flow rate (low and high), turbidity, pH, and nitrite had a negative effect on the number of embryos.The number of embryos was positively correlated with CS in nine of our 23 populations, but embryo number and PC1 were not related within any sample (Table S3).

## 4 DISCUSSION

*Potamopyrgus antipodarum* is well known for its extreme shell morphological variation and for the fact that asexually reproducing strains have successfully invaded diverse fresh and brackish water habitats around the globe. In this study, we aimed to establish whether phenotypic plasticity of morphology was a key factor to adapting to the novel habitats in Europe.

We detected only two mitochondrial 16S rRNA/cyt *b* haplotypes within all our samples: a widespread haplotype t/22 (92% of samples) and a rarer haplotype z2/37 (8%). This result is consistent with previous findings suggesting that Europe was colonized by two distinct 16S rRNA lineages, with the common haplotype t dominating in freshwaters and a rarer, predominantly brackish water haplotype *z* (Städler et al., 2005). Our haplotype z2 differed only by one mutation from haplotype *z*. Because the genetic sampling of European populations of *P. antipodarum* by both our and Städler et al.'s (2005) studies is still limited, it is difficult to assess whether this difference at one position reflects a systematic sequencing error due to the different methods applied, whether both lineages diverged after arrival in Europe, or whether we discovered a third mitochondrial lineage directly introduced from New Zealand, which we consider less likely. Additional sequencing effort both in Europe and New Zealand is needed to resolve this question. Cyt *b* haplotypes 22 and 37 and 16S haplotypes t and *z* were previously found in native *P. antipodarum* (Neiman & Lively, 2004; Neiman et al., 2011; Städler et al., 2005). It is still not possible to retrace the exact New Zealand region of origin for the European invasions because both cyt *b* haplotypes were found on both the North and South Island (Neiman & Lively, 2004; Neiman et al., 2011). Identifying the specific New Zealand source of the European invasion will require higher resolution on the nuclear level, using, for example, SNP markers.

We observed 10 SNP genotypes based on 16 variable loci. Genotypes 1–8 were present in mitochondrial haplotype t/22, and genotypes 9 and 10 were found in mitochondrial haplotype z2/37. Within haplotype t/22, genotype 1 was the most common (280 individuals, 80%) and widespread, present at all sites except for DERUG. Genotypes 2 to 7 only differed by a maximum of two mutations from genotype 1. While some of these genotypes had a relatively wide distribution, for example, genotypes 2 and 3 (Figure 2), most genotypes were found only in a single site. For example, genotype 4 only occurred at DEWIT, and genotypes 5 and 6 were only found at BEWIL. Genotype 8 (found only in DERUG) was exceptional in differing by seven substitutions from genotype 1. These findings and the central position of genotype 1 in the network analysis suggest that genotype 1 was the ancestral and originally introduced genotype, and that the other genotypes diverged during the roughly 180 years after the species' introduction, which is in accordance with Weetman et al.'s (2002) microsatellite‐based analysis. Within haplotype z2/37, we detected only two SNP genotypes, which is likely at least in part a consequence of the fact that we only genotyped 39 snails with this haplotype. Genotypes 9 (26 individuals, 67%) and 10 differed from each other by three mutations. Genotype 9 was found both in fresh and brackish sites, and genotype 10 only in brackish water. The presence of genetic variation within our sample means that we cannot rule out the possibility that the morphological variation that we observed was at least partly due to genetic adaptation instead of plasticity. Accordingly, we corrected for genetic lineage using haplotype and genotype nested within haplotype as random factors in our LMM and GLMMs. The remaining variation was therefore likely due to phenotypic plasticity, although we cannot exclude the existence of adaptive genetic variation below the resolution of our markers at loci influencing shell morphology. We consider this latter possibility unlikely because neutral mutations usually occur at a much higher rate than adaptive mutations and *P. antipodarum* invaded Europe only recently, within the last c. 180 years (Alonso & Castro‐Díez, 2012). And all but one genotype differed by only one or two mutations from the presumptive ancestral genotype. Nevertheless, conclusive evidence against adaptive genetic variation will require common garden or transplant experiments. While these experiments are simple in principle, the many environmental variables and populations involved means that they will be difficult to execute in practice. It is also worthwhile to emphasize the value that comes from analyzing unmanipulated, field‐collected individuals from natural populations.

We found a major genetic contribution to shell shape variation in *P. antipodarum*. Representatives of haplotype t/22 had on average a more slender and taller shape than the shorter but wider z2/37 individuals, with the widest shells observed at our only brackish site. This result is consistent with the previous categorization of European *P. antipodarum* into three distinct morphotypes (Warwick, 1952, 1969): a slender morphotype A later linked to Städler et al.'s (2005) haplotype t, and stouter morphotypes B and C linked to haplotype *z*. However, the difference in shape along PC1 was continuous, with some overlap of shells of both haplotypes. Hence, an unambiguous distinction of both haplotypes based on shape is not possible (see also the similar results from length–width ratios presented in Simpson, 1976). As the variation in shape was more pronounced between than within haplotypes, this variation probably reflected differentiation prior to the introduction to Europe whereas the variation within haplotypes was likely the result of postintroduction local adaptation. Latitude, temperature, and nitrate had direct effects on shell shape, nitrate also positively influenced brood size in slender snails, and flow rate and sunlight coverage interacted with shell shape on fecundity. Slender‐shelled snails carried more embryos than stouter individuals in high‐flow habitats. We observed the opposite pattern in habitats with no or low flow. Together, these results suggest that slender snails are better adapted to lotic habitats. More broadly, the fact that embryo number was negatively associated with increased flow rate indicates that rapid flow might be a stressor for *P. antipodarum*. Our field observations (see also Haase, 2003) suggest that snails avoid being exposed to strong flow by seeking shelter behind stones or in vegetation. In general, flow is a challenge for many small stream organisms because being detached and dragged away by the current can lead to physical damage and because flow can redeposit organisms in habitats that are less suitable than the original habitat (e.g., Holomuzki & Biggs, 1999, 2006). Without the constraint of flow, snails with wider shells produced more embryos than slender snails. This effect is increased by sunlight coverage. This result suggests that for *P. antipodarum*, wide shells are advantageous in the absence of current. The positive association between shell width and embryo production is probably linked to the fact that wider shells provide more space for the brood pouch and, thus, for more embryos (McKenzie et al., 2013).

The relation of shell shape, fitness, and flow rate is probably more complex than anticipated from previous studies showing that *P. antipodarum* living in habitats with rapid flow were wider and had a larger aperture than individuals collected in low flow conditions (Haase, 2003; Kistner & Dybdahl, 2014). We extended these studies by directly considering potential consequences for fitness by incorporating brood size. In general, the best‐adapted shape to flow for benthic invertebrates depends on organismal size and the flow rate that the organism encounters. Often, a relatively streamlined shape is more adaptive for relatively large individuals or for high‐flow conditions, while a relatively stout morph is more adaptive for smaller individuals or in lower flow rate (Vogel, 1994). These baseline expectations might provide a good explanation for our finding that slender snails had higher fitness in high flow. The complexity of shape adaptation to flow is partly due to the fact that a streamlined or dorsoventrally flattened shape may reduce drag but at the same time also increase lift (Vogel, 1994). The risk of being dislodged through lift instead of drag presumably increases with increasing velocities (Weissenberger, Emanns, & Schwoerbelt, 1985).

Aperture area relative to shell height increased with flow rate and is likely correlated with snail foot size and thus attachment area (Haase, 2003). The number of brooded embryos corrected for CS also increased with the relative aperture area for no and low flow. This increase was three times as important in the presence of low flow than in stagnant waters. Thus, increasing relative aperture size might represent an alternative strategy of attaining a relatively larger attachment area that is distinct from developing a wider shell shape and supports the hypothesis that in lotic environments, snails with a large aperture/foot are better adapted. In conclusion, with respect to flow, shell shape variation in *P. antipodarum* might be, at least in part, a function of the trade‐off reducing drag by having a slender and streamlined shell but still presenting a wide attachment area.

Apart from flow rate, sunlight coverage and nitrate concentration interacted with shell shape with respect to fecundity in our GLMM. Wide‐shelled snails had more embryos than slender‐shelled shells in environments with high sunlight coverage. The opposite relationship between shell width and fecundity was observed in habitats with higher nitrate concentration. Why a certain shell shape might be more adaptive in conjunction with these factors is unclear. Nitrogen compounds are naturally present in freshwater ecosystems through degradation processes of organic matter as bacteria oxidize ammonia to nitrite and nitrate (Stumm & Morgan, 1996). Anthropogenic sources can substantially increase the amount of nitrogen compounds in surface waters, which can in turn impact aquatic animals directly via toxicity (Jensen, 1995, 2003), or indirectly by decreasing the amount of dissolved oxygen as a result of eutrophication (Schindler et al., 1973). Among aquatic invertebrates, *P. antipodarum* exhibits relatively high tolerance to short‐term toxicity of nitrogen compounds (Alonso & Camargo, 2003). To us, it appears most plausible that sunlight coverage and nitrate concentration might influence *P. antipodarum* by affecting the composition and amount of available food sources such as algae and bacteria.

Similar to shell shape, shell smoothness had a strong genetic component apparent at the haplotype level (see also Hauser et al., 1992; Warwick, 1944, 1969), with the few ridged snails mostly harboring haplotype z2/37.

Our data suggest that shell size varies as a function of phenotypic plasticity. Like previous studies, we demonstrated a direct, positive association between size and fecundity in female *P. antipodarum* (McKenzie et al., 2013). Several environmental factors affected size or fecundity directly, but only two of these factors, flow and turbidity, had an effect on brood size in interaction with size.

The slope of the correlation between brood size and size was steeper in habitats with low flow compared to standing water bodies, suggesting that in lotic habitats, larger snails are better adapted than smaller ones. This result is consistent with previous findings in *P. antipodarum* (Haase, 2003), but is counter‐intuitive considering that the Reynolds number (Sommerfeld, 1908), predicting the transition from laminar to turbulent flow, increases with size (Statzner & Holm, 1989). Juvenile *P. antipodarum*, for instance, experience ~10‐times lower drag than the larger adults (Statzner, 1987). Here, we speculate that the relatively large forces that larger snails can exert for attachment to the substratum counterbalance the increase in drag. A similar phenomenon has been shown in amphipods (*Gammarus* sp.), where the energy contents of adult individuals compensated for the higher drag that they experienced relative to juveniles (Statzner, 1987). In habitats with high‐flow rate, brood size, and shell size were not correlated, again suggesting that current may be experienced as a stressor forcing reallocation of energies from fecundity. Finally, with respect to water turbidity, the correlation slope between size and embryos was steeper in clear water in comparison with turbid water, which may be linked to food availability.

Latitude was negatively associated with both shell and brood size, conductivity was positively associated with brood size, and sunlight coverage was positively associated with shell size. Latitude could perhaps better represent the annual mean temperature experienced by the snails than our point measurement at the time of collection. Numerous studies have shown that temperature and conductivity affect life history traits in *P. antipodarum* such as growth rate, fecundity, and survival (e.g., Bennett, Dudley, Cooper, & Sweet, 2014; Dybdahl & Kane, 2005; Gust et al., 2011; Herbst, Bogan, & Lusardi, 2008; Levri, Krist, Bilka, & Dybdahl, 2014; McKenzie et al., 2013). Conductivity can be linked to the presence of calcium, necessary for building the shell or producing embryos, but low ion concentrations may result in osmotic stress as well as increase the costs for calcium uptake (Herbst et al., 2008). The effects of the environmental factors nitrate, nitrite, turbidity, and pH on size and/or fecundity are probably best explained again via effects on food availability.

In contrast to shape, shell size did vary between DEHOB and DEHOT collected 1 year apart at the same site, suggesting that environmental conditions in one location can vary between years and result in a difference in shell size. Similarly, snails from the same lake were slightly larger at a depth of 2 m (DEJAT) compared to those collected near the surface (DEJAR) suggesting a depth‐related effect on size that did not involve adaptation to parasitism (see Vergara et al., 2016).

Shell size and number of embryos increased later in the season. Because fecundity in *P. antipodarum* increases with shell size, and because snails collected later in the year were larger than snails collected earlier in the year, we believe that this association of embryo number with timing was likely an incidental consequence of collection time of particular samples that had a larger adult size than samples collected earlier rather than a seasonal effect on embryo number itself.

## 5 CONCLUSIONS

Despite the presence of only two mitochondrial lineages among our 23 northwest European samples, we found that invasive *P. antipodarum* featured wide variation in both shell size and shape. The likely neutral genetic differentiation of asexual lineages detected using SNPs probably arose locally and post colonization. Variation in shell shape was most evident between the two mitochondrial lineages, suggesting that these lineages were already differentiated before their introduction to Europe c. 180 years ago. Local adaptation to invaded habitats, both genetic and plastic, was less important. By contrast, variation in shell size and fecundity (brood size) seemed to be driven mainly by plasticity. Incorporating brood size as a proxy for fitness, we revealed that the plastic responses of shell morphology to environmental parameters were more complex than hypothesized.

Generally speaking, flow appears to be a stressor constraining fecundity. However, in stagnant waters, snails with large and broad shells had relatively large brood sizes. By contrast, larger, rather slender snails with a larger relative aperture area seem to be better adapted to high flow habitats. Shape, size, and fecundity were also influenced by other abiotic parameters. In particular, physicochemical factors potentially affecting food availability had an effect on fecundity, partly in conjunction with shell morphology. Our findings are largely based on a few lineages, and therefore, we cannot exclude the possibility that other lineages show different responses (cf. Levri & Clark, 2015).

Long‐term monitoring of environmental parameters would improve the basis for studying adaptive processes. Information on interactions with predators (e.g., Levri, 1998) will also further improve our understanding of the adaptive function of shell morphology, which has been demonstrated in other aquatic gastropods such as *Littorina obtusata* (Seeley, 1986), *Elimia livescens* (Krist, 2002), or *Physa* sp. (DeWitt, Robinson, & Wilson, 2000). At this point, we can conclude first that shell morphology in *P. antipodarum* is likely to vary at least in part as an adaptation to specific environmental factors, including stressful conditions. This adaptability might play a key role in *P. antipodarum*'s successful invasion of a broad range of habitats; and secondly, that the variation of the two main components of morphology, namely shape and size, are differentially controlled, the former mainly genetically and the latter predominantly by phenotypic plasticity.

## CONFLICT OF INTEREST

None declared.

## AUTHOR CONTRIBUTIONS

Design and conception, fieldwork, data analysis, and laboratory work: GV and MH. Genetics and manuscript: GV, KEM, LB, MN, and MH.

## DATA ACCESSIBILITY

Morphological and environmental data are available on the Dryad Digital Repository (DOI: https://doi.org/10.5061/dryad.93464f7). The newly developed SNP markers are available on the European Variation Archive (accession: PRJEB24869)and the 16S mitochondrial DNA sequence z2 on GenBank (accession: MG581815).

## References

[ece34009-bib-0001] Alonso, Á. , & Camargo, J. A. (2003). Short‐term toxicity of ammonia, nitrite, and nitrate to the aquatic snail *Potamopyrgus antipodarum* (Hydrobiidae, Mollusca). Bulletin of Environment Contamination and Toxicology, 70, 1006–1012. https://doi.org/10.1007/s00128-003-0082-5 10.1007/s00128-003-0082-5 12719828

[ece34009-bib-0002] Alonso, Á. , & Castro‐Díez, P. (2008). What explains the invading success of the aquatic mud snail *Potamopyrgus antipodarum* (Hydrobiidae, Mollusca)? Hydrobiologia, 614, 107–116. https://doi.org/10.1007/s10750-008-9529-3

[ece34009-bib-0003] Alonso, Á. , & Castro‐Díez, P. (2012). The exotic aquatic mud snail *Potamopyrgus antipodarum* (Hydrobiidae, Mollusca): State of the art of a worldwide invasion. Aquatic Sciences, 74, 375–383. https://doi.org/10.1007/s00027-012-0254-7

[ece34009-bib-0004] Bankers, L. A. , Fields, P. , McElroy, K. E. , Boore, J. L. , Logsdon, J. M. , & Neiman, M. (2017). Genomic evidence for population‐specific responses to coevolving parasites in a New Zealand freshwater snail. Molecular Ecology, 26, 3663–3675. https://doi.org/10.1111/mec.14146 28429458 10.1111/mec.14146

[ece34009-bib-0005] Bartoń, K. (2017). MuMIn: Multi‐model inference. R package version 1.40.0. Retrieved from https://CRAN.R-project.org/package=MuMIn

[ece34009-bib-0006] Bates, D. , Maechler, M. , Bolker, B. , & Walker, S. (2015). Fitting linear mixed‐effects models using lme4. Journal of Statistical Software, 67(1), 1–48.

[ece34009-bib-0007] Becker, R. A. , Chambers, J. M. , & Wilks, A. R. (1988). The new S language. Pacific Grove, CA: Wadsworth & Brooks.

[ece34009-bib-0008] Bennett, D. M. , Dudley, T. L. , Cooper, S. D. , & Sweet, S. S. (2014). Ecology of the invasive New Zealand mud snail, *Potamopyrgus antipodarum* (Hydrobiidae), in a mediterranean‐climate stream system. Hydrobiologia, 746, 375–399.

[ece34009-bib-0009] Bookstein, F. L. (1991). Morphometric tools for landmark data. Cambridge, UK: Cambridge University Press.

[ece34009-bib-0010] Bowler, P. A. (1991). The rapid spread of the freshwater hydrobiid snail *Potamopyrgus antipodarum* (Gray) in the Middle Snake River, southern Idaho. Proceedings of the Desert Fishes Council, 21, 173–182.

[ece34009-bib-0011] Carja, O. , Liberman, U. , & Feldman, M. W. (2014). Evolution in changing environments: Modifiers of mutation, recombination, and migration. Proceedings of the National Academy of Sciences of the United States of America, 111, 17935–17940. https://doi.org/10.1073/pnas.1417664111 25427794 10.1073/pnas.1417664111 PMC4273399

[ece34009-bib-0012] Charmantier, A. , McCleery, R. H. , Cole, L. R. , Perrins, C. M. , Kruuk, L. E. B. , & Sheldon, B. C. (2008). Adaptive phenotypic plasticity in response to climate change in a wild bird population. Science, 320, 800–803. https://doi.org/10.1126/science.1157174 18467590 10.1126/science.1157174

[ece34009-bib-0013] Chevin, L. M. , Lande, R. , & Mace, G. M. (2010). Adaptation, plasticity, and extinction in a changing environment: Towards a predictive theory. PLoS Biology, 8(4), e1000357. 20463950 10.1371/journal.pbio.1000357 PMC2864732

[ece34009-bib-0014] Collado, G. A. (2014). Out of New Zealand: Molecular identification of the highly invasive freshwater mollusk *Potamopyrgus antipodarum* (Gray, 1843) in South America. Zoological Studies, 53, 1–9.

[ece34009-bib-0015] Dawson, T. P. , Jackson, S. T. , House, J. I. , Prentice, I. C. , & Mace, G. M. (2011). Beyond predictions: Biodiversity conservation in a changing climate. Science, 332(6025), 53–58. https://doi.org/10.1126/science.1200303 21454781 10.1126/science.1200303

[ece34009-bib-0016] DeWitt, T. J. , Robinson, B. W. , & Wilson, D. S. (2000). Functional diversity among predators of a freshwater snail imposes an adaptive trade‐off for shell morphology. Evolutionary Ecology Research, 2, 129–148.

[ece34009-bib-0017] DeWitt, T. J. , Sih, A. , & Wilson, D. S. (1998). Costs and limits of phenotypic plasticity. Trends in Ecology & Evolution, 13, 77–81. https://doi.org/10.1016/S0169-5347(97)01274-3 21238209 10.1016/s0169-5347(97)01274-3

[ece34009-bib-0018] Dybdahl, M. F. , & Kane, S. L. (2005). Adaptation vs. phenotypic plasticity in the success of a clonal invader. Ecological Society of America: Issues In Ecology, 86, 1592–1601.

[ece34009-bib-0019] Dybdahl, M. F. , & Lively, C. M. (1995). Diverse, endemic and polyphyletic clones in mixed populations of a freshwater snail (*Potamopyrgus antipodarum*). Journal of Evolutionary Biology, 8, 385–398. https://doi.org/10.1046/j.1420-9101.1995.8030385.x

[ece34009-bib-0020] Fox, J. (2003). Effect displays in R for generalised linear models. Journal of Statistical Software, 8, 1–27.

[ece34009-bib-0021] Fox, J. , & Weisberg, S. (2011). An R companion to applied regression, 2nd ed Thousand Oaks CA: Sage.

[ece34009-bib-0022] Gangloff, M. M. (1998). The New Zealand mud snail in Western North America. Aquatic Nuisance Species, 2, 25–30.

[ece34009-bib-0023] Gérard, C. , & Le Lannic, J. (2003). Establishment of a new host–parasite association between the introduced invasive species *Potamopyrgus antipodarum* (Smith) (Gastropoda) and *Sanguinicola* sp. Plehn (Trematoda) in Europe. Journal of Zoology, 261(2), 213–216. https://doi.org/10.1017/S0952836903004084

[ece34009-bib-0024] Ghalambor, C. K. , McKay, J. K. , Carroll, S. P. , & Reznick, D. N. (2007). Adaptive versus non‐adaptive phenotypic plasticity and the potential for contemporary adaptation in new environments. Functional Ecology, 21, 394–407. https://doi.org/10.1111/j.1365-2435.2007.01283.x

[ece34009-bib-0025] Gienapp, P. , Teplitsky, C. , Alho, J. S. , Mills, J. A. , & Merilä, J. (2008). Climate change and evolution: Disentangling environmental and genetic responses. Molecular Ecology, 17, 167–178. https://doi.org/10.1111/j.1365-294X.2007.03413.x 18173499 10.1111/j.1365-294X.2007.03413.x

[ece34009-bib-0026] Gingerich, P. D. (2009). Rates of evolution. Annual Review of Ecology Evolution and Systematics, 40, 657–675. https://doi.org/10.1146/annurev.ecolsys.39.110707.173457

[ece34009-bib-0027] Gittenberger, E. , Piel, W. H. , & Groenenberg, D. S. J. (2004). The Pleistocene glaciations and the evolutionary history of the polytypic snail species *Arianta arbustorum* (Gastropoda, Pulmonata, Helicidae). Molecular Phylogenetics and Evolution, 30, 64–73. https://doi.org/10.1016/S1055-7903(03)00182-9 15022758 10.1016/s1055-7903(03)00182-9

[ece34009-bib-0028] Gust, M. , Buronfosse, T. , André, C. , Mons, R. , Gagné, F. , & Garric, J. (2011). Is exposure temperature a confounding factor for the assessment of reproductive parameters of New Zealand mudsnails *Potamopyrgus antipodarum* (Gray)? Aquatic Toxicology, 101, 396–404. https://doi.org/10.1016/j.aquatox.2010.11.013 21216350 10.1016/j.aquatox.2010.11.013

[ece34009-bib-0029] Gust, M. , Gagné, F. , Berlioz‐Barbier, A. , Besse, J. P. , Buronfosse, T. , Tournier, M. , … Cren‐Olivé, C. (2014). Caged mudsnail *Potamopyrgus antipodarum* (Gray) as an integrated field biomonitoring tool: Exposure assessment and reprotoxic effects of water column contamination. Water Research, 54, 222–236. https://doi.org/10.1016/j.watres.2014.01.057 24576698 10.1016/j.watres.2014.01.057

[ece34009-bib-0030] Haase, M. (2003). Clinal variation in shell morphology of the freshwater gastropod *Potamopyrgus antipodarum* along two hill‐country streams in New Zealand. Journal of the Royal Society of New Zealand, 33, 549–560. https://doi.org/10.1080/03014223.2003.9517743

[ece34009-bib-0031] Haase, M. (2008). The radiation of hydrobiid gastropods in New Zealand: A revision including the description of new species based on morphology and mtDNA sequence information. Systematics and Biodiversity, 6, 99–159. https://doi.org/10.1017/S1477200007002630

[ece34009-bib-0032] Hammer, Ø. , Harper, D. A. T. , & Ryan, P. D. (2001). PAST: Paleontological statistics software package for education and data analysis. Palaeontologia Electronica, 4(1), 9.

[ece34009-bib-0033] Hauser, L. , Carvalho, G. R. , Hughes, R. N. , & Carter, R. E. (1992). Clonal structure of the introduced freshwater snail *Potamopyrgus antipodarum* (Prosobranchia: Hydrobiidae), as revealed by DNA fingerprintring. Proceedings of the Royal Society B: Biological Sciences, 249, 19–25. https://doi.org/10.1098/rspb.1992.0078 1359547 10.1098/rspb.1992.0078

[ece34009-bib-0034] Hechinger, R. F. (2012). Faunal survey and identification key for the trematodes (Platyhelminthes: Digenea) infecting *Potamopyrgus antipodarum* (Gastropoda: Hydrobiidae) as first intermediate host. Zootaxa, 27, 1–27.

[ece34009-bib-0035] Hendry, A. P. , Farrugia, T. J. , & Kinnison, M. T. (2008). Human influences on rates of phenotypic change in wild animal populations. Molecular Ecology, 17, 20–29. https://doi.org/10.1111/j.1365-294X.2007.03428.x 18173498 10.1111/j.1365-294X.2007.03428.x

[ece34009-bib-0036] Herbst, D. B. , Bogan, M. T. , & Lusardi, R. A. (2008). Low specific conductivity limits growth and survival of the New Zealand mud snail from the Upper Owens River, California. Western North American Naturalist, 68, 324–333. https://doi.org/10.3398/1527-0904(2008)68[324:LSCLGA]2.0.CO;2

[ece34009-bib-0037] Holomuzki, J. R. , & Biggs, B. J. F. (1999). Distributional responses to flow disturbance by a stream‐dwelling snail. Oikos, 87, 36–47. https://doi.org/10.2307/3546994

[ece34009-bib-0038] Holomuzki, J. R. , & Biggs, B. J. F. (2006). Habitat‐specific variation and performance trade‐offs in shell armature of New Zealand mudsnails. Ecological Society of America: Issues In Ecology, 87, 1038–1047. 10.1890/0012-9658(2006)87[1038:hvapti]2.0.co;2 16676547

[ece34009-bib-0039] Hughes, R. N. (1996). Evolutionary ecology of parthenogenetic strains of the prosobranch snail*, Potamopyrgus antipodarum* (Gray)= *P. jenkinsi* (Smith). Malacological Review, 28, 101–114.

[ece34009-bib-0040] Jacobsen, R. , Forbes, V. E. , & Skovgaard, O. (1996). Genetic population structure of the prosobranch snail *Potamopyrgus antipodarum* (Gray) in Denmark using PCR‐RAPD fingerprints. Proceedings of the Royal Society B: Biological Sciences, 263, 1065–1070. https://doi.org/10.1098/rspb.1996.0157 8805840 10.1098/rspb.1996.0157

[ece34009-bib-0041] Jensen, F. B. (1995). Uptake and effects of nitrite and nitrate in animals. Nitrogen Metabolism and Excretion. Boca Raton, FL: CRC Press.

[ece34009-bib-0042] Jensen, F. B. (2003). Nitrite disrupts multiple physiological functions in aquatic animals. Comparative Biochemistry and Physiology Part A Molecular Integrative Physiology, 135, 9–24. https://doi.org/10.1016/S1095-6433(02)00323-9 10.1016/s1095-6433(02)00323-9 12727546

[ece34009-bib-0043] Johnson, P. C. (2014). Extension of Nakagawa & Schielzeth's *R* ^2^ _GLMM_ to random slopes models. Methods in Ecology and Evolution, 5(9), 944–946. https://doi.org/10.1111/2041-210X.12225 25810896 10.1111/2041-210X.12225 PMC4368045

[ece34009-bib-0044] Jokela, J. , Dybdahl, M. F. , & Lively, C. M. (1999). Habitat‐specific variation in life‐history traits, clonal population structure and parasitism in a freshwater snail (*Potamopyrgus antipodarum*). Journal of Evolutionary Biology, 12, 350–360. https://doi.org/10.1046/j.1420-9101.1999.00035.x

[ece34009-bib-0045] Jokela, J. , & Lively, C. M. (1995a). Parasites, sex, and early reproduction in a mixed population of freshwater snails. Evolution, 49, 1268–1271. https://doi.org/10.1111/j.1558-5646.1995.tb04453.x 28568513 10.1111/j.1558-5646.1995.tb04453.x

[ece34009-bib-0046] Jokela, J. , & Lively, C. M. (1995b). Spatial variation in infection by digenetic trematodes in a population of freshwater snails (*Potamopyrgus antipodarum*). Oecologia, 103, 509–517. https://doi.org/10.1007/BF00328690 28307000 10.1007/BF00328690

[ece34009-bib-0047] Jokela, J. , Lively, C. M. , Dybdahl, M. F. , & Fox, J. A. (1997). Evidence for a cost of sex in the freshwater snail *Potamopyrgus antipodarum* . Ecology, 78, 452–460. https://doi.org/10.1890/0012-9658(1997)078[0452:EFACOS]2.0.CO;2

[ece34009-bib-0048] Kistner, E. J. , & Dybdahl, M. F. (2013). Adaptive responses and invasion: The role of plasticity and evolution in snail shell morphology. Ecology and Evolution, 3, 424–436. https://doi.org/10.1002/ece3.471 23467920 10.1002/ece3.471 PMC3586651

[ece34009-bib-0049] Kistner, E. J. , & Dybdahl, M. F. (2014). Parallel variation among populations in the shell morphology between sympatric native and invasive aquatic snails. Biological Invasions, 16, 2615–2626. https://doi.org/10.1007/s10530-014-0691-4

[ece34009-bib-0050] Klingenberg, C. P. (2011). MorphoJ: An integrated software package for geometric morphometrics. Molecular Ecology Resources, 11, 353–357. https://doi.org/10.1111/j.1755-0998.2010.02924.x 21429143 10.1111/j.1755-0998.2010.02924.x

[ece34009-bib-0051] Klingenberg, C. P. (2016). Size, shape, and form: Concepts of allometry in geometric morphometrics. Development Genes and Evolution, 226, 113–137. https://doi.org/10.1007/s00427-016-0539-2 27038023 10.1007/s00427-016-0539-2 PMC4896994

[ece34009-bib-0052] Klingenberg, C. P. , & Monteiro, L. R. (2005). Distances and directions in multidimensional shape spaces: Implications for morphometric applications. Systematic Biology, 54, 678–688. https://doi.org/10.1080/10635150590947258 16126663 10.1080/10635150590947258

[ece34009-bib-0053] Krist, A. C. (2002). Crayfish induce a defensive shell shape in a freshwater snail. Invertebrate Biology, 121, 235–242.

[ece34009-bib-0054] Lagrue, C. , McEwan, J. , Poulin, R. , & Keeney, D. B. (2007). Co‐occurrences of parasite clones and altered host phenotype in a snail‐trematode system. International Journal for Parasitology, 37, 1459–1467. https://doi.org/10.1016/j.ijpara.2007.04.022 17582419 10.1016/j.ijpara.2007.04.022

[ece34009-bib-0055] Langmead, B. , & Salzberg, S. L. (2013). Fast grapped‐read alignment with Bowtie 2. Nature Methods, 9(4), 357–359. 10.1038/nmeth.1923 PMC3322381 22388286

[ece34009-bib-0056] Levri, E. P. (1998). Perceived predation risk, parasitism, and the foraging behavior of a freshwater snail (*Potamopyrgus antipodarum*). Canadian Journal of Zoology, 76, 1878–1884. https://doi.org/10.1139/z98-122

[ece34009-bib-0057] Levri, E. P. , & Clark, T. J. (2015). Behavior in invasive New Zealand mud snails (*Potamopyrgus antipodarum*) is related to source population. Biological Invasions, 17, 497–506. https://doi.org/10.1007/s10530-014-0746-6

[ece34009-bib-0058] Levri, E. P. , Dillard, J. , & Martin, T. (2005). Trematode infection correlates with shell shape and defence morphology in a freshwater snail. Parasitology, 130, 699–708. https://doi.org/10.1017/S0031182005007286 15977907 10.1017/s0031182005007286

[ece34009-bib-0059] Levri, E. P. , Krist, A. C. , Bilka, R. , & Dybdahl, M. F. (2014). Phenotypic plasticity of the introduced New Zealand mud snail, *Potamopyrgus antipodarum*, compared to sympatric native snails. PLoS ONE, 9(4), e93985 https://doi.org/10.1371/journal.pone.0093985 24699685 10.1371/journal.pone.0093985 PMC3974863

[ece34009-bib-0060] Li, H. , Handsaker, B. , Wysoker, A. , Fennell, T. , Ruan, J. , Homer, N. , … Durbin, R. (2009). The sequence alignment/map format and SAMtools. Bioinformatics, 25, 2078–2079. https://doi.org/10.1093/bioinformatics/btp352 19505943 10.1093/bioinformatics/btp352 PMC2723002

[ece34009-bib-0061] Lively, C. M. (1987). Evidence from a New Zealand snail for the maintenance of sex by parasitism. Nature, 329, 855–857.3313054

[ece34009-bib-0062] Matthiessen, P. (2008). An assessment of endocrine disruption in mollusks and the potential for developing internationally standardized mollusk life cycle test guidelines. Integrated Environmental Assessment and Management, 4, 274–284. https://doi.org/10.1897/IEAM_2008-003.1 18393578 10.1897/IEAM_2008-003.1

[ece34009-bib-0063] McKenzie, V. J. , Hall, W. E. , & Guralnick, R. P. (2013). New Zealand mudsnails (*Potamopyrgus antipodarum*) in Boulder Creek, Colorado: Environmental factors associated with fecundity of a parthenogenic invader. Canadian Journal of Zoology, 91, 30–36. https://doi.org/10.1139/cjz-2012-0183

[ece34009-bib-0064] Mergeay, J. , Verschuren, D. , & De Meester, L. (2006). Invasion of an asexual American water flea clone throughout Africa and rapid displacement of a native sibling species. Proceedings of the Royal Society B: Biological Sciences, 273, 2839–2844. https://doi.org/10.1098/rspb.2006.3661 17015310 10.1098/rspb.2006.3661 PMC1664637

[ece34009-bib-0065] Moloney, K. A. , Holzapfel, C. , Tielbörger, K. , Jeltsch, F. , & Schurr, F. M. (2009). Rethinking the common garden in invasion research. Perspectives in Plant Ecology, Evolution and Systematics, 11, 311–320. https://doi.org/10.1016/j.ppees.2009.05.002

[ece34009-bib-0066] Monteiro, L. R. (1999). Multivariate regression models and geometric morphometrics: The search for causal factors in the analysis of shape. Systematic Biology, 48, 192–199. https://doi.org/10.1080/106351599260526 12078640 10.1080/106351599260526

[ece34009-bib-0067] Nakagawa, S. , & Schielzeth, H. (2013). A general and simple method for obtaining *R* ^2^ from generalized linear mixed‐effects models. Methods in Ecology and Evolution, 4, 133–142. https://doi.org/10.1111/j.2041-210x.2012.00261.x

[ece34009-bib-0068] Negovetic, S. , & Jokela, J. (2001). Life‐history variation, phenotypic plasticity and maintenance of subpopulation structure in a freshwater snail. Ecology, 82, 2805–2815. https://doi.org/10.1890/0012-9658(2001)082[2805:LHVPPA]2.0.CO;2

[ece34009-bib-0069] Neiman, M. , Larkin, K. , Thompson, A. R. , & Wilton, P. (2012). Male offspring production by asexual *Potamopyrgus antipodarum*, a New Zealand snail. Heredity (Edinburgh), 109, 57–62. https://doi.org/10.1038/hdy.2012.13 22491063 10.1038/hdy.2012.13 PMC3375405

[ece34009-bib-0070] Neiman, M. , & Lively, C. M. (2004). Pleistocene glaciation is implicated in the phylogeographical structure of *Potamopyrgus antipodarum*, a New Zealand snail. Molecular Ecology, 13, 3085–3098. https://doi.org/10.1111/j.1365-294X.2004.02292.x 15367122 10.1111/j.1365-294X.2004.02292.x

[ece34009-bib-0071] Neiman, M. , & Lively, C. M. (2005). Male New Zealand mud snails (*Potamopyrgus antipodarum*) persist in copulating with asexual and parasitically castrated females. American Midland Naturalist, 154, 88–96. https://doi.org/10.1674/0003-0031(2005)154[0088:MNZMSP]2.0.CO;2

[ece34009-bib-0072] Neiman, M. , Paczesniak, D. , Soper, D. M. , Baldwin, A. T. , & Hehman, G. (2011). Wide variation in ploidy level and genome size in a new zealand freshwater snail with coexisting sexual and asexual lineages. Evolution, 65, 3202–3216. https://doi.org/10.1111/j.1558-5646.2011.01360.x 22023586 10.1111/j.1558-5646.2011.01360.x

[ece34009-bib-0073] Paczesniak, D. , Jokela, J. , Larkin, K. , & Neiman, M. (2013). Discordance between nuclear and mitochondrial genomes in sexual and asexual lineages of the freshwater snail *Potamopyrgus antipodarum* . Molecular Ecology, 22, 4695–4710. https://doi.org/10.1111/mec.12422 23957656 10.1111/mec.12422

[ece34009-bib-0074] Ponder, W. F. (1988). *Potamopyrgus antipodarum*: A molluscan colonizer of Europe and Australia. Journal of Molluscan Studies, 54, 271–286. https://doi.org/10.1093/mollus/54.3.271

[ece34009-bib-0075] R Development Core Team (2011). R: A language and environment for statistical computing. Vienna, Austria: R foundation for Statistical Computing,.

[ece34009-bib-0076] Reznick, D. N. (2001). The population ecology of contemporary adaptations: What empirical. Genetica, 112, 183–198. https://doi.org/10.1023/A:1013352109042 11838765

[ece34009-bib-0077] Rohlf, F. J. (2010). TpsDig, Version 2.16. Department of Ecology and Evolution, State University of New York at Stony Brook, USA. Retrieved from http://life.bio.sunysb.edu/morph/bibr28

[ece34009-bib-0078] Rohlf, F. J. (2012). TpsUtil, File Utility Program. Version 1.53. Department of Ecology and Evolution, State University of New York at Stony Brook, USA. Retrieved from http://life.bio.sunysb.edu/morph

[ece34009-bib-0079] Rohlf, F. J. , & Slice, D. (1990). Extensions of the Procrustes method for the optimal superimposition of landmarks. Systematic Biology, 39, 40–59.

[ece34009-bib-0080] Sakai, A. K. , Allendorf, F. W. , Holt, J. S. , Lodge, D. M. , Molofsky, J. , Baughman, S. , … Weller, S. G. (2001). The population biology of invasive species. Annual Review of Ecology and Systematics, 32, 305–332. https://doi.org/10.1146/annurev.ecolsys.32.081501.114037

[ece34009-bib-0081] Salamin, N. , Wüest, R. O. , Lavergne, S. , Thuiller, W. , & Pearman, P. B. (2010). Assessing rapid evolution in a changing environment. Trends in Ecology & Evolution, 25, 692–698. https://doi.org/10.1016/j.tree.2010.09.009 20961648 10.1016/j.tree.2010.09.009

[ece34009-bib-0082] Schilthuizen, M. , & Haase, M. (2010). Disentangling true shape differences and experimenter bias: Are dextral and sinistral snail shells exact mirror images ? Journal of Zoology, 282, 191–200. https://doi.org/10.1111/j.1469-7998.2010.00729.x 21258640 10.1111/j.1469-7998.2010.00729.x PMC3020325

[ece34009-bib-0083] Schindler, D. W. , Kling, H. , Schmidt, R. V. , Prokopowich, J. , Frost, V. E. , Reid, R. A. , & Capel, M. (1973). Eutrophication of Lake 227 by addition of phosphate and nitrate: The second, third, and fourth years of enrichment, 1970, 1971, and 1972. Journal of the Fisheries Board of Canada, 30, 1415–1440. https://doi.org/10.1139/f73-233

[ece34009-bib-0084] Schreiber, E. S. G. , Glaister, A. , Quinn, G. P. , & Lake, P. S. (1998). Life history and population dynamics of the exotic snail Potamopyrgus antipodarum (Prosobranchia :Hydrobiidae) in Lake Purrumbete, Victoria, Australia. Marine & Freshwater Research, 49, 73–78. https://doi.org/10.1071/MF97113

[ece34009-bib-0085] Seeley, R. H. (1986). Intense natural selection caused a rapid morphological transition in a living marine snail. Proceedings of the National Academy of Sciences of the United States of America, 83, 6897–6901. https://doi.org/10.1073/pnas.83.18.6897 16593760 10.1073/pnas.83.18.6897 PMC386617

[ece34009-bib-0086] Sheets, D. H. (2011). IMP software. Department of Physics, Canisius College, Buffalo, NY, Department of Geology, SUNY at Buffalo, Buffalo, NY, USA. Retrieved from http://www3.canisius.edu/~sheets/IMP%208.htm

[ece34009-bib-0087] Shimada, K. , & Urabe, M. (2003). Comparative ecology of the alien freshwater snail *Potamopyrgus antipodarum* and the indigenous snail Semisulcospira spp. Venus, 62, 39–53.

[ece34009-bib-0088] Sieratowicz, A. , Stange, D. , Schulte‐Oehlmann, U. , & Oehlmann, J. (2011). Reproductive toxicity of bisphenol A and cadmium in *Potamopyrgus antipodarum* and modulation of bisphenol A effects by different test temperature. Environmental Pollution, 159, 2766–2774. https://doi.org/10.1016/j.envpol.2011.05.012 21737193 10.1016/j.envpol.2011.05.012

[ece34009-bib-0089] Simpson, J. F. (1976). On the existence of discrete morphological types within the Species *Potamopyrgus jenkinsi* (Smith). Journal of Molluscan Studies, 42, 108–113.

[ece34009-bib-0090] Skole, D. , & Tucker, C. (1993). Evidence for tropical deforestation, fragmented habitat, and adversely affected habitat in the Brazilian Amazon: 1978–1988. Science, 260, 1905–1910. https://doi.org/10.1126/science.260.5116.1905 17836720 10.1126/science.260.5116.1905

[ece34009-bib-0091] Smith, J. , Schneider, S. , Oppenheimer, M. , Yohe, G. , Hare, W. , Mastrandrea, M. , … van Ypersele, J.‐P. (2009). Assessing dangerous climate change through an update of the Intergovernmental Panel on Climate Change (IPCC) “reasons for concern”. Proceedings of the National Academy of Sciences of the United States of America, 106, 4133–4137. https://doi.org/10.1073/pnas.0812355106 19251662 10.1073/pnas.0812355106 PMC2648893

[ece34009-bib-0092] Sommerfeld, A. (1908). Ein beitrag zur hydrodynamischen erklaerung der turbulenten fluessigkeitsbewegungen. Atti del IV Congresso Internazionale dei Matematici, 4, 116–124.

[ece34009-bib-0093] Städler, T. , Frye, M. , Neiman, M. , & Lively, C. M. (2005). Mitochondrial haplotypes and the New Zealand origin of clonal European *Potamopyrgus*, an invasive aquatic snail. Molecular Ecology, 14, 2465–2473. https://doi.org/10.1111/j.1365-294X.2005.02603.x 15969728 10.1111/j.1365-294X.2005.02603.x

[ece34009-bib-0094] Statzner, B. (1987). Ökologische Bedeutung der sohlennahen Strömungsgeschwindigkeit für benthische Wirbellose in Fließgewässern. Doctoral dissertation, Thesis, Univ. Karlsruhe, Germany

[ece34009-bib-0095] Statzner, B. , & Holm, T. F. (1989). Morphological adaptation of shape to flow: Microcurrents around lotic macroinvertebrates with known Reynolds numbers at quasi‐natural flow conditions. Oecologia, 78, 145–157. https://doi.org/10.1007/BF00377150 28312353 10.1007/BF00377150

[ece34009-bib-0096] Stearns, S. C. (1989). The evolutionary significance of phenotypic plasticity. BioScience, 39, 436–445. https://doi.org/10.2307/1311135

[ece34009-bib-0500] Strong, E. E. , Gargominy, O. , Ponder, W. F. , & Bouchet, P. (2008). Global diversity of gastropods (Gastropoda; Mollusca) in freshwater. Hydrobiologia, 595, 149–166. https://doi.org/10.1007/s10750-007-9012-6

[ece34009-bib-0097] Stumm, W. , & Morgan, J. J. (1996). Aquatic chemistry, 3rd ed New York, NY: John Wiley Sons.

[ece34009-bib-0098] Sultan, S. (1995). Phenotypic plasticity and plant adaptation. Acta Botanica Neerlandica, 44, 363–383. https://doi.org/10.1111/j.1438-8677.1995.tb00793.x

[ece34009-bib-0099] Symanowski, F. , & Hildebrandt, J. P. (2010). Differences in osmotolerance in freshwater and brackish water populations of *Theodoxus fluviatilis* (Gastropoda: Neritidae) are associated with differential protein expression. Journal of Comparative Physiology B, 180, 337–346. https://doi.org/10.1007/s00360-009-0435-4 10.1007/s00360-009-0435-4 20012055

[ece34009-bib-0100] Vergara, D. , Fuentes, J. A. , Stoy, K. S. , & Lively, C. M. (2016). Evaluating shell variation across different populations of a freshwater snail. Molluscan Research, 5818, 1–13.

[ece34009-bib-0102] Vogel, S. (1994). Life in moving fluids: The physical biology of flow. Princeton, NJ: Princeton University Press.

[ece34009-bib-0103] Völker, C. , Gräf, T. , Schneider, I. , Oetken, M. , & Oehlmann, J. (2014). Combined effects of silver nanoparticles and 17α‐ethinylestradiol on the freshwater mudsnail *Potamopyrgus antipodarum* . Environmental Science and Pollution Research, 21, 10661–10670. https://doi.org/10.1007/s11356-014-3067-5 24888616 10.1007/s11356-014-3067-5

[ece34009-bib-0104] Vonesh, E. F. , Chinchilli, V. M. , & Pu, K. (1996). Goodness‐of‐fit in generalized nonlinear mixed‐effects models. Biometrics, 52(2), 572–587. https://doi.org/10.2307/2532896 10766504

[ece34009-bib-0105] Wallace, C. (1992). Parthenogenesis, sex and chromosomes in *Potamopyrgus* . Journal of Molluscan Studies, 58, 93–107. https://doi.org/10.1093/mollus/58.2.93

[ece34009-bib-0106] Warwick, T. (1944). Inheritance of the keel in *Potamopyrgus jenkinsi* (Smith). Nature, 154, 798–799. https://doi.org/10.1038/154798b0

[ece34009-bib-0107] Warwick, T. (1952). Strains in the mollusc *Potamopyrgus jenkinsi* (Smith). Nature, 169, 551–552. https://doi.org/10.1038/169551a0 14929240 10.1038/169551a0

[ece34009-bib-0108] Warwick, T. (1969). Systematics of the genus *Potamopyrgus* (Hydrobiidae) in Europe, and the causation of the keel in this snail. Malacologia, 9, 301–302.

[ece34009-bib-0109] Weetman, D. , Hauser, L. , & Carvalho, G. R. (2002). Reconstruction of microsatellite mutation history reveals a strong and consistent deletion bias in invasive clonal snails, *Potamopyrgus antipodarum* . Genetics, 162, 813–822. 12399391 10.1093/genetics/162.2.813 PMC1462296

[ece34009-bib-0110] Weissenberger, J. , Emanns, H. S. A. , & Schwoerbelt, J. (1985). Measurement of lift and drag forces in the mN range experienced by benthic arthropods at flow velocities below 1. 2 m s^−1^ . Freshwater Biology, 25, 21–31.

[ece34009-bib-0111] Winterbourn, M. J. (1970a). Population studies on the New Zealand freshwater gastropod *Potamopyrgus antipodarum* (gray). Journal of Molluscan Studies, 39, 139–149. https://doi.org/10.1093/oxfordjournals.mollus.a065088

[ece34009-bib-0112] Winterbourn, M. J. (1970b). The New Zealand species of *Potamopyrgus* (Gastropoda: Hydrobiidae). Malacologia, 10, 283–321.

[ece34009-bib-0113] Winterbourn, M. J. (1974). Larval Trematoda parasitizing the New Zealand species of *Potamopyrgus* (Gastropoda: Hydrobiidae). Mauri Ora, 2, 17–30.

[ece34009-bib-0114] Xie, D. , Yu, D. , Yu, L. F. , & Liu, C. H. (2010). Asexual propagations of introduced exotic macrophytes *Elodea nuttallii*,* Myriophyllum aquaticum*, and *M. propinquum* are improved by nutrient‐rich sediments in China. Hydrobiologia, 655, 37–47. https://doi.org/10.1007/s10750-010-0402-9

[ece34009-bib-0115] Zbikowski, J. , & Zbikowska, E. (2009). Invaders of an invader ‐ trematodes in *Potamopyrgus antipodarum* in Poland. Journal of Invertebrate Pathology, 101, 67–70. https://doi.org/10.1016/j.jip.2009.02.005 19249307 10.1016/j.jip.2009.02.005

[ece34009-bib-0116] Zelditch, M. L. , Swiderski, D. L. , & Sheets, H. D. (2012). Geometric morphometrics for biologists: A primer. Waltham, MA: Academic Press.

