# Supplementary material for: Adaptive phenotypic plasticity in a clonal invader
Source: Ecol Evol. 2018 Apr 2;8(9):4465–83. doi: 10.1002/ece3.4009 (PMC5938463; doi:10.1002/ece3.4009)
Supplement: Supplementary file 1 [file ECE3-8-4465-s001.docx]

Appendix tables

**Appendix table 1** SNP-IDs and polymorphic comparison between the SNP validation trial and the European populations of *P. antipodarum*. SNP- IDs starting with “comp” were newly developed (EVA accession:  PRJEB24869), IDs starting with “ss” were previously developed by Paczesniak *et al.* (2013). P = polymorphic, F = fixed across all samples.

| SNP ID | Trial | Europe |
| --- | --- | --- |
| comp128041_c0_seq2 | F | F |
| comp129781_c0_seq2 | P | not genotyped |
| comp132525_c0_seq1 | F | P |
| comp134834_c0_seq1 | F | F |
| comp135714_c0_seq12 | F | F |
| comp136417_c0_seq1 | F | not genotyped |
| comp136626_c0_seq3 | P | not genotyped |
| comp138809_c0_seq1 | P | not genotyped |
| comp140766_c3_seq1 | P | P |
| comp141103_c0_seq2 | P | P |
| comp141987_c0_seq1 | P | P |
| comp142905_c0_seq3 | P | not genotyped |
| comp143011_c0_seq1 | P | F |
| comp143177_c2_seq1 | F | F |
| comp144295_c3_seq1 | P | P |
| comp145273_c3_seq2 | F | not genotyped |
| comp146173_c0_seq1 | P | F |
| comp146583_c0_seq2 | P | P |
| comp146704_c0_seq1 | F | F |
| comp148591_c0_seq1 | P | F |
| comp149246_c0_seq5 | F | F |
| comp149304_c0_seq1 | P | F |
| comp149357_c1_seq35 | F | F |
| comp149466_c1_seq1 | P | not genotyped |
| comp149840_c0_seq1 | P | not genotyped |
| comp150035_c0_seq4 | P | F |
| comp150764_c0_seq1 | P | not genotyped |
| comp150837_c0_seq1 | P | not genotyped |
| comp150869_c0_seq1 | P | not genotyped |
| comp151037_c0_seq2 | F | F |
| comp151129_c0_seq1 | F | not genotyped |
| comp152324_c0_seq1 | P | not genotyped |
| comp152737_c0_seq2 | P | P |
| comp152808_c0_seq4 | P | not genotyped |
| comp152973_c0_seq1 | P | P |
| comp153021_c1_seq35 | F | genotyping failed |
| comp153035_c0_seq1 | P | genotyping failed |
| comp153594_c0_seq11 | P | F |
| comp154585_c0_seq1 | P | F |
| comp154607_c0_seq5 | P | not genotyped |
| comp157372_c0_seq6 | F | F |
| comp157769_c0_seq1 | P | F |
| comp158084_c2_seq1 | P | not genotyped |
| comp159303_c0_seq1 | F | F |
| comp159857_c0_seq1 | P | not genotyped |
| comp160076_c0_seq1 | F | genotyping failed |
| comp160266_c0_seq4 | P | F |
| comp160355_c0_seq15 | F | not genotyped |
| comp160897_c0_seq2 | F | F |
| comp161222_c2_seq1 | P | not genotyped |
| comp161255_c0_seq3 | F | F |
| comp161323_c0_seq4 | F | F |
| comp161499_c2_seq1 | P | not genotyped |
| comp162159_c1_seq8 | P | not genotyped |
| comp162353_c0_seq1 | P | not genotyped |
| comp162441_c0_seq1 | F | F |
| comp162551_c0_seq3 | P | not genotyped |
| comp162701_c0_seq4 | P | P |
| comp162799_c1_seq1 | P | not genotyped |
| comp163375_c0_seq1 | F | not genotyped |
| comp163630_c0_seq1 | P | P |
| comp163685_c0_seq1 | P | genotyping failed |
| comp163987_c0_seq1 | F | F |
| comp184712_c0_seq1 | F | not genotyped |
| ss804270584 | not genotyped | P |
| ss804270586 | not genotyped | genotyping failed |
| ss804270587 | not genotyped | F |
| ss804270588 | not genotyped | F |
| ss804270589 | not genotyped | genotyping failed |
| ss804270590 | not genotyped | F |
| ss804270592 | not genotyped | F |
| ss804270593 | not genotyped | genotyping failed |
| ss804270594 | not genotyped | F |
| ss804270595 | not genotyped | P |
| ss804270596 | not genotyped | P |
| ss804270598 | not genotyped | P |
| ss804270599 | not genotyped | F |
| ss804270601 | not genotyped | genotyping failed |
| ss804270603 | not genotyped | F |
| ss804270605 | not genotyped | P |
| ss804270606 | not genotyped | F |
| ss804270607 | not genotyped | genotyping failed |
| ss804270608 | not genotyped | genotyping failed |
| ss804270610 | not genotyped | F |
| ss804270611 | not genotyped | genotyping failed |
| ss804270614 | not genotyped | P |
| ss804270615 | not genotyped | F |

| - | BEBRA | BEGER | BEHER | BEKAS | BEOOE | BEOOT | BEVEU | BEWIL | DEBIN | DEDOB | DEHOB | DEHOT | DEJAR | DEJAT | DEPAS | DERUG | DESEG | DESEL | DEWEL | DEWER | DEWIT | NL1 | NL2 |
| --- | --- | --- | --- | --- | --- | --- | --- | --- | --- | --- | --- | --- | --- | --- | --- | --- | --- | --- | --- | --- | --- | --- | --- |
| BEBRA | - | 0.0315 | 0.0208 | 0.0234 | 0.0329 | 0.0367 | 0.0258 | 0.0368 | 0.0536 | 0.0535 | 0.0427 | 0.0349 | 0.0321 | 0.0207 | 0.0377 | 0.0486 | 0.0356 | 0.0349 | 0.0270 | 0.0332 | 0.0400 | 0.0265 | 0.0446 |
| BEGER | 0.0017 | - | 0.0248 | 0.0289 | 0.0388 | 0.0205 | 0.0356 | 0.0508 | 0.0656 | 0.0430 | 0.0319 | 0.0285 | 0.0329 | 0.0289 | 0.0335 | 0.0301 | 0.0294 | 0.0297 | 0.0260 | 0.0282 | 0.0350 | 0.0330 | 0.0403 |
| BEHER | 0.2184 | 0.2117 | - | 0.0172 | 0.0294 | 0.0232 | 0.0280 | 0.0485 | 0.0649 | 0.0434 | 0.0332 | 0.0254 | 0.0285 | 0.0234 | 0.0316 | 0.0369 | 0.0294 | 0.0282 | 0.0198 | 0.0284 | 0.0334 | 0.0293 | 0.0465 |
| BEKAS | 0.2150 | 0.1982 | 0.8534 | - | 0.0326 | 0.0294 | 0.0296 | 0.0444 | 0.0657 | 0.0409 | 0.0306 | 0.0230 | 0.0285 | 0.0207 | 0.0313 | 0.0406 | 0.0317 | 0.0291 | 0.0174 | 0.0311 | 0.0324 | 0.0286 | 0.0461 |
| BEOOE | **0.0002** | **<.0001** | 0.0069 | 0.0118 | - | 0.0313 | 0.0264 | 0.0541 | 0.0780 | 0.0398 | 0.0320 | 0.0328 | 0.0366 | 0.0399 | 0.0289 | 0.0404 | 0.0317 | 0.0260 | 0.0281 | 0.0232 | 0.0298 | 0.0338 | 0.0535 |
| BEOOT | **<.0001** | 0.0633 | 0.0226 | 0.0058 | **<.0001** | - | 0.0346 | 0.0592 | 0.0782 | 0.0359 | 0.0251 | 0.0244 | 0.0340 | 0.0361 | 0.0284 | 0.0261 | 0.0286 | 0.0252 | 0.0236 | 0.0241 | 0.0299 | 0.0377 | 0.0497 |
| BEVEU | 0.0122 | **0.0002** | 0.0250 | 0.0543 | 0.0037 | **<.0001** | - | 0.0376 | 0.0639 | 0.0489 | 0.0352 | 0.0308 | 0.0263 | 0.0311 | 0.0347 | 0.0474 | 0.0295 | 0.0329 | 0.0258 | 0.0317 | 0.0357 | 0.0251 | 0.0400 |
| BEWIL | **0.0001** | **<.0001** | **0.0001** | 0.0022 | **<.0001** | **<.0001** | **0.0001** | **-** | 0.0495 | 0.0668 | 0.0558 | 0.0485 | 0.0447 | 0.0356 | 0.0560 | 0.0668 | 0.0499 | 0.0535 | 0.0449 | 0.0519 | 0.0573 | 0.0423 | 0.0419 |
| DEBIN | **<.0001** | **<.0001** | **<.0001** | **<.0001** | **<.0001** | **<.0001** | **<.0001** | **<.0001** | **-** | 0.0917 | 0.0805 | 0.0725 | 0.0603 | 0.0535 | 0.0807 | 0.0806 | 0.0671 | 0.0750 | 0.0662 | 0.0752 | 0.0811 | 0.0548 | 0.0473 |
| DEDOB | **<.0001** | **<.0001** | **<.0001** | **0.0002** | **<.0001** | **<.0001** | **<.0001** | **<.0001** | **<.0001** | **-** | 0.0223 | 0.0289 | 0.0485 | 0.0469 | 0.0223 | 0.0362 | 0.0335 | 0.0243 | 0.0322 | 0.0303 | 0.0214 | 0.0460 | 0.0573 |
| DEHOB | **<.0001** | 0.0003 | 0.0036 | 0.0441 | **0.0002** | **0.0002** | **<.0001** | **<.0001** | **<.0001** | 0.0155 | **-** | 0.0148 | 0.0313 | 0.0371 | 0.0219 | 0.0254 | 0.0218 | 0.0198 | 0.0191 | 0.0237 | 0.0231 | 0.0337 | 0.0440 |
| DEHOT | **<.0001** | 0.0018 | 0.0210 | 0.1335 | **<.0001** | **0.0002** | **<.0001** | **<.0001** | **<.0001** | **<.0001** | 0.3956 | **-** | 0.0241 | 0.0266 | 0.0256 | 0.0300 | 0.0227 | 0.0230 | 0.0149 | 0.0270 | 0.0270 | 0.0283 | 0.0408 |
| DEJAR | 0.0010 | **<.0001** | 0.0039 | 0.0242 | **<.0001** | **<.0001** | 0.0031 | **<.0001** | **<.0001** | **<.0001** | **0.0001** | 0.0010 | **-** | 0.0261 | 0.0407 | 0.0366 | 0.0251 | 0.0369 | 0.0242 | 0.0382 | 0.0421 | 0.0199 | 0.0322 |
| DEJAT | 0.0456 | 0.0016 | 0.0910 | 0.3835 | **<.0001** | **<.0001** | 0.0008 | **0.0001** | **<.0001** | **<.0001** | **<.0001** | 0.0018 | 0.0019 | **-** | 0.0366 | 0.0446 | 0.0303 | 0.0345 | 0.0233 | 0.0357 | 0.0384 | 0.0246 | 0.0383 |
| DEPAS | **<.0001** | **<.0001** | 0.0008 | 0.0143 | 0.0003 | **<.0001** | **<.0001** | **<.0001** | **<.0001** | 0.0040 | 0.0248 | 0.0009 | **<.0001** | **<.0001** | - | 0.0374 | 0.0253 | 0.0122 | 0.0222 | 0.0163 | 0.0081 | 0.0357 | 0.0512 |
| DERUG | **<.0001** | 0.0026 | **0.0002** | 0.0005 | **<.0001** | **0.0001** | **<.0001** | **<.0001** | **<.0001** | **<.0001** | 0.0086 | **0.0001** | **<.0001** | **<.0001** | **<.0001** | **-** | 0.0290 | 0.0318 | 0.0312 | 0.0317 | 0.0387 | 0.0401 | 0.0459 |
| DESEG | **0.0001** | 0.0004 | 0.0021 | 0.0072 | **<.0001** | **<.0001** | 0.0003 | **<.0001** | **<.0001** | **<.0001** | 0.0214 | 0.0023 | 0.0005 | **<.0001** | 0.0015 | **0.0002** | - | 0.0216 | 0.0176 | 0.0234 | 0.0258 | 0.0226 | 0.0336 |
| DESEL | **<.0001** | **0.0002** | 0.0044 | 0.0154 | 0.0009 | **<.0001** | **<.0001** | **<.0001** | **<.0001** | 0.0005 | 0.0454 | 0.0031 | **<.0001** | **<.0001** | 0.4892 | **<.0001** | 0.0051 | **-** | 0.0173 | 0.0119 | 0.0119 | 0.0312 | 0.0452 |
| DEWEL | 0.0009 | 0.0031 | 0.1216 | 0.4409 | **0.0001** | 0.0004 | 0.0011 | **<.0001** | **<.0001** | **<.0001** | 0.0474 | 0.1828 | 0.0009 | 0.0040 | 0.0062 | **<.0001** | 0.0351 | 0.0519 | - | 0.0203 | 0.0230 | 0.0225 | 0.0380 |
| DEWER | **<.0001** | 0.0004 | 0.0021 | 0.0079 | 0.0046 | **<.0001** | **<.0001** | **<.0001** | **<.0001** | **<.0001** | 0.0052 | **0.0001** | **<.0001** | **<.0001** | 0.1065 | **0.0001** | 0.0007 | 0.5313 | 0.0046 | - | 0.0181 | 0.0336 | 0.0471 |
| DEWIT | **<.0001** | **<.0001** | **<.0001** | 0.0075 | 0.0003 | **<.0001** | **<.0001** | **<.0001** | **<.0001** | 0.0058 | 0.0112 | **<.0001** | **<.0001** | **<.0001** | 0.9513 | **<.0001** | 0.0004 | 0.5530 | 0.0024 | 0.0394 | - | 0.0357 | 0.0512 |
| NL1 | 0.0061 | **0.0002** | 0.0137 | 0.0712 | **<.0001** | **<.0001** | 0.0137 | **<.0001** | **<.0001** | **<.0001** | **0.0002** | 0.0007 | 0.0460 | 0.0109 | **<.0001** | **<.0001** | 0.0144 | 0.0003 | 0.0102 | **<.0001** | **0.0001** | - | 0.0295 |
| NL2 | **<.0001** | **<.0001** | **<.0001** | **<.0001** | **<.0001** | **<.0001** | **<.0001** | **<.0001** | **<.0001** | **<.0001** | **<.0001** | **<.0001** | **0.0001** | **<.0001** | **<.0001** | **<.0001** | **<.0001** | **<.0001** | **<.0001** | **<.0001** | **<.0001** | 0.0017 | **-** |

**Appendix table 2** Pairwise Procrustes distances. Distances above diagonal, *P-*values below; significance (α = 0.00029 after Bonferroni correction) indicated in bold

**Appendix table 3** Relationships of brood size and shell morphology within populations.

| Population | Correlation | CS | | PC1 | |
| --- | --- | --- | --- | --- | --- |
|  |  | **Statistic** | ***P*-value** | **Statistic** | ***P*-value** |
| BEBRA | Spearman's rs | 0.2451 | 0.3270 | 0.0507 | 0.8417 |
| BEBRA | Spearman's rs | 0.2451 | 0.3270 | 0.0507 | 0.8417 |
| BEGER | Pearson's r | 0.5721 | 0.0131 | -0.0704 | 0.7914 |
| BEHER | Spearman's rs | 0.2256 | 0.5308 | 0.4451 | 0.1974 |
| BEKAS | Pearson's r | 0.5383 | 0.2126 | 0.6171 | 0.1399 |
| BEOOE | Spearman's rs | 0.2691 | 0.2801 | -0.0714 | 0.7782 |
| BEOOT | Pearson's r | 0.441 | 0.0516 | -0.0911 | 0.7024 |
| BEVEU | Pearson's r | 0.5691 | 0.0088 | -0.4032 | 0.0779 |
| BEWIL | Pearson's r | 0.59616 | 0.0055 | 0.1345 | 0.5719 |
| DEBIN | Pearson's r | 0.8387 | 0.0048 | -0.4346 | 0.2424 |
| DEDOB | Spearman's rs | 0.01056 | 0.9648 | -0.0060 | 0.9798 |
| DEHOB | Pearson's r | 0.4028 | 0.1365 | 0.6190 | 0.0139 |
| DEHOT | Pearson's r | 0.3264 | 0.2173 | -0.1961 | 0.4836 |
| DEJAR | Spearman's rs | 0.4470 | 0.0482 | 0.0500 | 0.8342 |
| DEJAT | Pearson's r | 0.5658 | 0.0093 | 0.3337 | 0.1505 |
| DEPAS | Pearson's r | 0.7256 | 0.0003 | -0.2080 | 0.3788 |
| DERUG | Pearson's r | 0.1807 | 0.5030 | 0.0722 | 0.7903 |
| DESEG | Pearson's r | 0.3100 | 0.1835 | 0.0098 | 0.9673 |
| DESEL | Pearson's r | 0.5953 | 0.0056 | 0.2460 | 0.3250 |
| DEWEL | Pearson's r | 0.4002 | 0.0804 | 0.0040 | 0.9867 |
| DEWER | Pearson's r | 0.4701 | 0.0365 | -0.0604 | 0.8003 |
| DEWIT | Pearson's r | 0.5229 | 0.0213 | 0.2131 | 0.3812 |
| NL1 | Pearson's r | 0.4152 | 0.0771 | -0.3451 | 0.1479 |
| NL2 | Pearson's r | 0.4360 | 0.1043 | -0.3056 | 0.2680 |
